# CD9 inhibition reveals a functional connection of extracellular vesicle secretion with mitophagy in melanoma cells

**DOI:** 10.1002/jev2.12082

**Published:** 2021-05-12

**Authors:** Henar Suárez, Zoraida Andreu, Carla Mazzeo, Víctor Toribio, Aldo Emmanuel Pérez‐Rivera, Soraya López‐Martín, Susana García‐Silva, Begoña Hurtado, Esperanza Morato, Laura Peláez, Egoitz Astigarraga Arribas, Tarson Tolentino‐Cortez, Gabriel Barreda‐Gómez, Ana Isabel Marina, Héctor Peinado, María Yáñez‐Mó

**Affiliations:** ^1^ Departamento de Biología Molecular Universidad Autónoma de Madrid (UAM) Madrid Spain; ^2^ Centro de Biología Molecular Severo Ochoa Instituto de Investigación Sanitaria La Princesa (IIS‐IP) Madrid Spain; ^3^ Facultad de Ciencias Universidad Nacional Autónoma de México, (UNAM), México City México; ^4^ Spanish National Cancer Research Centre (CNIO) Madrid Spain; ^5^ Unidad de Proteómica CBM‐SO CSIC‐UAM Madrid Spain; ^6^ IMG Pharma Biotech S.L. Parque Tecnológico de Zamudio Derio Spain

**Keywords:** CD9, cytopermeable peptides, EV biogenesis, mitophagy, tetraspanin

## Abstract

Tetraspanins are often used as Extracellular Vesicle (EV) detection markers because of their abundance on these secreted vesicles. However, data on their function on EV biogenesis are controversial and compensatory mechanisms often occur upon gene deletion. To overcome this handicap, we have compared the effects of tetraspanin CD9 gene deletion with those elicited by cytopermeable peptides with blocking properties against tetraspanin CD9. Both CD9 peptide or gene deletion reduced the number of early endosomes. CD9 peptide induced an increase in lysosome numbers, while CD9 deletion augmented the number of MVB and EV secretion, probably because of compensatory CD63 expression upregulation. *In vivo*, CD9 peptide delayed primary tumour cell growth and reduced metastasis size. These effects on cell proliferation were shown to be concomitant with an impairment in mitochondrial quality control. CD9 KO cells were able to compensate the mitochondrial malfunction by increasing total mitochondrial mass reducing mitophagy. Our data thus provide the first evidence for a functional connection of tetraspanin CD9 with mitophagy in melanoma cells.

## 1 INTRODUCTION

Extracellular Vesicles (EVs) have emerged as a potent mechanism of intercellular communication that can contain a complex message composed of several signalling factors, enzymatic activities and genetic material (Yáñez‐Mó et al., 2015). They are present in all biological fluids, offering a great opportunity for non‐invasive biomarker discovery in several clinical scenarios; and have therapeutic potential for a wide variety of diseases, including neurodegenerative disorders and cancer (Fais et al., 2016).

Tetraspanins are commonly used as markers of EVs populations, being present on both microvesicles (EVs budding from the plasma membrane) and exosomes (EVs derived from late endosomal multivesicular bodies (MVB) compartments) (Andreu & Yáñez‐Mó, 2014). However, no fundamental alterations in endosome biogenesis and maturation have been described in tetraspanin KO mice, except for CD63, which is crucial for the formation of melanosomes in melanocytes (Van Niel et al., 2011). Data on how tetraspanin expression can modulate the number of exosomes released by the cell is still controversial; while CD9 or CD63 gene deletion have been shown to decrease EV numbers (Chairoungdua et al., 2010; Hurwitz et al., 2016), in other reports no differences were observed (Brzozowski et al., 2018; Perez‐Hernandez et al., 2013). Tetraspanins have been proposed to be preferentially involved in the ESCRT‐independent route for EV biogenesis (Van Niel et al., 2011), although they interact with components of the ESCRT complexes: CD63 with Syntenin‐1 (Latysheva et al., 2006) or CD9 with Alix (Romancino et al., 2018). In contrast, there is ample evidence of tetraspanin control of the intracellular trafficking of their associated molecules throughout the different endolysosomal compartments (Berditchevski & Odintsova, 2007; Rocha‐Perugini et al., 2017; Termini & Gillette, 2017). Tetraspanins also have an important role in the inclusion of the cargo within EVs, so that tetraspanin gene deletion selectively affects the recruitment of a set of tetraspanin‐associated molecules to exovesicles (Brzozowski et al., 2018; Perez‐Hernandez et al., 2013).

The endolysosomal system is closely imbricated with cellular metabolism. Lysosomes are capable of sensing the cellular NAD^+^/NADH ratio and adapting their biogenesis to the cellular metabolic state. Mitochondrial respiratory chain decoupling agents generate a transient increase in lysosomal biogenesis (Audano et al., 2018), while mitochondrial genetic defects reduce lysosomal biogenesis and fusion with autophagosomes (Baixauli et al., 2015). Several endosomal compartments are a source of membrane for autophagosome formation (Bissa & Deretic, 2018; Davis et al., 2017) and secretory autophagy supposes the release of extracellular vesicles (Xu et al., 2018). Thus, the endosomal system is a key node in the metabolic regulation of the cell.

There is no tetraspanin‐null cell in higher eukaryotes, and upon gene deletion, compensatory mechanisms commonly occur, evidenced by the very severe phenotype of double tetraspanin KO mice (Jin et al., 2018) compared to the usually mild or cell type specific effect of single tetraspanin deletion (Deng et al., 2002; Kelić et al., 2001; Le Naour, 2000; Miyado, 2000). To overcome these difficulties we have designed cell‐penetrating guanidinium‐rich peptides (Stanzl et al., 2013) containing the C‐terminal sequence of tetraspanins. C‐terminal region was demonstrated to be fundamental for tetraspanin‐enriched microdomains connection with intracellular signalling and cytoskeletal components (Perez‐Hernandez et al., 2013). TEM intracellular interactome was shown to be quite complex (Perez‐Hernandez et al., 2013), and included several adaptor molecules such as ERMs (Sala‐Valdés et al., 2006), actinin (Gordón‐Alonso et al., 2012) and filamin (Perez‐Hernandez et al., 2013) among others. Many interactions were overlapping between different tetraspanins and tetraspanin‐associated receptors, while some others were found to be tetraspanin‐specific (Perez‐Hernandez et al., 2013; Rocha‐Perugini et al., 2017). We have previously reported that treatment of cells with these cell‐penetrating peptides mimics many of the effects observed upon tetraspanin silencing or gene deletion. Thus, CD81 peptide impairs Rac‐dependent cell migration (Tejera et al., 2013), TNFα secretion (Martínez Del Hoyo et al., 2015) and HIV‐1 reverse transcription (Rocha‐Perugini et al., 2017). Peptides for CD63 or CD151 impair papilloma and cytomegalovirus infection (Fast et al., 2018). Here we have studied the effects of CD9 cytopermeable peptides on EV secretion in a melanoma model. Our data suggest a fundamental role for this tetraspanin in regulating cellular metabolism by affecting early endosome formation and mitophagy in melanoma cells.

## 2 MATERIALS AND METHODS

### 2.1 Ab initio modelling

For ab initio modelling, short sequences were modelled with QUARK Software, which builds the 3D structure using the replica‐exchange method of Monte‐Carlo and guided at atomic level (Xu & Zhang, 2013). To assemble the fragments into a complete structure we employed the extended C‐QUARK version, that integrates Deep‐Learning strategies to predict the best contacts among the modelled fragments (Zhang et al., 2018). This structure was then further refined with ModRefine software. To produce a protein‐lipid membrane system we employed CHARMM‐GUI with a specific lipid composition which included 60% LPC, 30% POPC and 10% of endosomal lipids as well as a solution containing Na+ and Cl‐ ions to solvate the interior and exterior surfaces (Wu et al., 2014). The thickness and orientation of the membrane was adjusted to the membrane limits generated by OPM PPSERVER (Lomize et al., 2012) according to the chosen lipid composition. All resulting structures had a Tm‐SCORE ≥ 0.5. Complete structures were validated by Protein Software Analysis(ProSA) (Wiederstein & Sippl, 2007) and Ramachandra plots (RAMPAGE Tool) (Lovell et al., 2003). For modelling guanidinium‐rich peptides we used the PEP FOLD 3.5 tool based on Coarse‐grain force field coupling with and structural alphabet (Camproux et al., 2004; Lamiable et al., 2016). To gain power of the tool we adjusted the parameter to generate 200 simulations and selected from the best 10 clusters structure only those with the lowest free energy.

### 2.2 Cell culture

The human melanoma cell line SK‐MEL‐147 was cultured in DMEM supplemented with penicillin (100 U/ml), streptomycin (100 μg/ml) and 10% EV‐depleted FBS in a 5% CO_2_ humidified atmosphere at 37°C. FBS was depleted of bovine EVs by ultracentrifugation at 100,000 g for 16 h. For EV production, 1.6 10^6^ SK‐MEL‐147 cells were seeded in p150 plates and left in culture during 7d until they reached confluence, around 20 10^6^ cells/dish. Treatment with cytopermeable peptides was performed every 48 h along 7d using peptides at a final concentration of 5 μM in culture medium.

### 2.3 Reagents and constructs

Tetramethylrhodamine (TAMRA) N‐terminal–labelled peptides with the sequences RRRRRRRCCAIRRNREMV (CD9), RRRRRRRCCLVKSIRSGYEVM (CD63), or RRRRRRRYSVNICRGCSS (Scrambled) were purchased from LifeTein (South Plainfield).

For CD9 CRISPR/Cas9 gene deletion the pX461 plasmid was used inserting at the Bsb1 restriction site the guide RNA sequences directed to exon 2: Guide1: FW 5′ CACCGTTCTTGCTCGAAGATGCTCT‐3′; Guide2 FW 5′ CACCGGAATCGGAGCCATAGTCCAA‐3′. Transfected cells were sorted to be positive for GFP expression 48 h post‐transfection, kept in culture for one week and, after staining with specific anti‐CD9 antibodies (VJ1/20), selection of negative cells was again performed by sorting using a FACSAria Fusion (Becton Dickinson). Gene deletion was confirmed by flow cytometry and western‐blot.

To monitor autophagy flow, we used the mTagRFP‐mWasabi‐LC3 construct courtesy of Tamotsu Yoshimori (Osaka University). Mt‐mKeima construct was used to analyse mitophagy in living cells (Klionsky et al., 2016) and was kindly provided by Ana María Cuervo (Albert Einstein College of Medicine).

Antibodies used were anti‐CD9 (clone VJ1/20) and anti‐CD63 (clone Tea3/10) (Yáñez‐Mó et al., 1998), anti‐CD81 5A6 (provided by Dr S Levy, Stanford USA), anti‐ERM 90.3 rabbit polyclonal (provided by Dr. H Furthmayr, Stanford, USA), anti‐EEA1 (610457, BD Biosciences), anti‐HGS (ab72053, Abcam), antí‐LAMP1 (H4A3, Abcam), anti‐p62 (Cell Signalling Technology), anti‐LC3 (4E12, MBL international), Ki67 FLEX (mouse monoclonal clone MIB‐IR626, DAKO); CD31 (rabbit polyclonal ab28364, Abcam) or MPO (rabbit polyclonal A0398, DAKO), anti‐Syntaxin‐4 (610440, BD Biosciences), anti‐Syntenin‐1 (133003, Synaptic Systems), anti‐Flotillin (610821, BD), anti‐Tenascin (ab19011, Abcam), anti‐Nucleolin (H‐250, sc‐13057, Santa Cruz), anti‐Clathrin heavy chain (ab21679, Abcam), anti‐Rac1 (23A8)(05‐389, Sigma‐Aldrich), anti‐ATP5B (Rabbit polyclonal) (provided by Dr. JM Cuezva, CBMSO, Madrid, Spain)..

### 2.4 Isolation of extracellular vesicles

We have submitted all relevant data of our experiments to the EV‐TRACK knowledgebase (EV‐TRACK ID: EV210041) (Van Deun et al., 2017).

#### 2.4.1 Size exclusion chromatography (SEC)

A total of 45 ml of conditioned culture medium from 3 confluent p150 per condition (corresponding to 60 10^6^ cells with a viability over 98%) was subjected to centrifugations at 400 g for 5 min and 10 min at 2000 g to remove cells and cell debris. The resulting medium was concentrated by ultrafiltration at 2000 g for 30 minutes using Amicon Ultra‐15 filters (100K, Millipore, Billerica MA), to obtain a final volume of 1.5 ml which was loaded onto a SEC column (70 nm^+^ (IZON). 20 fractions of 500 μl were collected by gravity elution with filtered PBS. Detection of the EV‐enriched fractions was performed by dot‐blot or bead‐assisted flow cytometry as described (Suárez et al., 2017), with anti‐CD63 (TEA3/10) mAb. The three fractions (usually 8th‐10th) with the highest intensity were pooled for subsequent analyses.

#### 2.4.2 Ultracentrifugation

Conditioned culture medium was centrifuged at 400 g for 5 min and at 2000 g for 10 min to remove cells and cell debris. The supernatant was centrifuged again at 17000 g for 20 min and then at 100000 g for 2 h in a Sorvall AH‐627 rotor ultracentrifuge, L8‐70 M, (Beckman). The pellet obtained was washed with 30 ml of PBS and centrifuged again for 2 h at 100000 g. The final pellet was resuspended in 1.5 ml of PBS.

#### 2.4.3 Nanoparticle tracking analysis (NTA)

The concentration of EVs was determined using the NANOSIGHT LM10 (Malvern Instruments Ltd, Malvern, UK) equipped with a camera charge coupled device (CCD) (model F‐033) and a 638 nm laser. Analyses were made using NTA 3.0 software. The detection threshold was adjusted to 5. 60 s videos repeated in triplicate were recorded with the camera shutter at 30.02 ms and gain to 650, as recommended by the manufacturer.

### 2.5 Proteomic analyses

#### 2.5.1 In solution digestion

After denaturation of protein with 8 M urea in 50 mM ammonium bicarbonate pH 8.8, the sample was reduced and alkylated: with 10 mM DTT for 1 h at 37 °C, and then with 10 mM iodoacetamide for 30 min at room temperature in darkness. The sample was diluted to reduce urea concentration below 1.4 M and digested using sequencing grade trypsin (Promega, Madison, WI) overnight at 37° C using a 1:20 (w/w) enzyme:protein ratio. Digestion was stopped by the addition of 1% TFA. Whole supernatants were dried down and then desalted onto OMIX Pipette tips C18 (Agilent Technologies) (Torres et al., 2013).

#### 2.5.2 TMT labelling and high pH fractionation

The resultant peptide mixture (50 μg) was labelled using chemicals from the TMT sixplex Isobaric Mass Tagging Kit (Thermo Fisher Scientific, MA, USA) essentially as described by the manufacturer. Briefly, peptides were dissolved in 50 *μ*l of 100 mM triethylammonium bicarbonate (TEAB), adjusted to pH 8. For labelling, each TMT reagent was dissolved in 41 *μ*l of acetonitrile and added to the respective peptide mixture and then incubated at room temperature for one hour. Labelling was stopped by the addition of 8 *μ*l 5% hydroxylamine. Whole supernatants were dried down and the six samples were mixed to obtain the “6plex‐labeled mixture” (Zhou et al., 2019). The mixture was analysed by RP‐LC‐MS/MS to check the labelling efficiency.

The sample was then fractionated using the Pierce High pH Reversed‐Phase Peptide Fractionation Kitt (Thermo Fisher Scientific, MA, USA): sample was re‐swollen in 0.1%TFA and loaded onto an equilibrated, high‐pH, reversed‐phase fractionation spin column. A step gradient of increasing acetonitrile concentrations (5%–80%) in a volatile high‐pH (Triethylamine (0.1%)) was then applied to the columns to elute bound peptides into nine different fractions collected by centrifugation, dried and stored until analysis.

#### 2.5.3 Quantitative analysis by reverse phase‐liquid chromatography RP‐LC‐MS/MS

The fractions were resuspended in 10 μl of 0.1% formic acid and analysed by RP‐LC‐MS/MS in an Easy‐nLC II system coupled to an ion trap LTQ‐Orbitrap‐Velos‐Pro hybrid mass spectrometer (Thermo Scientific) (Clement et al., 2018). The peptides were concentrated (on‐line) by reverse phase chromatography using a 0.1 mm × 20 mm C18 RP precolumn (Thermo Scientific), and then separated using a 0.075 mm x 250 mm C18 RP column (Thermo Scientific) operating at 0.3 μl/min. Peptides were eluted using a 90‐min dual gradient. The gradient profile was set as follows: 5%−25% solvent B for 68 min, 25%−40% solvent B for 22 min, 40%−100% solvent B for 2 min and 100% solvent B for 18 min (Solvent A: 0,1% formic acid in water, solvent B: 0,1% formic acid, 80% acetonitrile in water). ESI ionization was done using a Nano‐bore emitters Stainless Steel ID 30 μm (Proxeon) interface at 2.1 kV spray voltage with S‐Lens of 60%.

The instrument method consisted of a data‐dependent top‐20 experiment with an Orbitrap MS1 scan at a resolution (*m*/Δ*m*) of 30,000 followed by either twenty high energy collision dissociation (HCD) MS/MS mass‐analyzed in the Orbitrap at 7500 (Δ*m*/*m*) resolution. MS2 experiments were performed using HCD to generate high resolution and high mass accuracy MS2 spectra. The minimum MS signal for triggering MS/MS was set to 500. The lock mass option was enabled for both MS and MS/MS mode and the polydimethylcyclosiloxane ions (protonated (Si(CH3)2O))6; *m*/*z* 445.120025) were used for internal recalibration of the mass spectra. Peptides were detected in survey scans from 400 to 1600 amu (1 μscan) using an isolation width of 1.3 u (in mass‐to‐charge ratio units), normalized collision energy of 40% for HCD fragmentation, and dynamic exclusion applied during 60 s periods. Charge‐state screening was enabled to reject unassigned and singly charged protonated ions.

#### 2.5.4 Quantitative data analysis

Peptide identification from raw data (a single search was performed with all nine raws from the fractionation) was carried out using PEAKS Studio X**+** search engine (Bioinformatics Solutions Inc., Waterloo, Ontario, Canada). Database search was performed against uniprot‐homo‐sapiens.fasta (74811 entries; UniProt release 12/2019) (decoy‐fusion database). The following constraints were used for the searches: tryptic cleavage after Arg and Lys (semispecific), up to two missed cleavage sites, and tolerances of 20 ppm for precursor ions and 0.05 Da for MS/MS fragment ions, and allowing optional Met oxidation and Cys carbamidomethylation and fixed TMT 6plex reagent labelling at the N‐terminus and lysine residues (Zhou et al., 2019). False discovery rates (FDR) for peptide spectrum matches (PSM) was limited to 0.01. Only those proteins with at least two distinct peptides and at least one unique peptides being discovered from LC/MS/MS analyses were considered reliably identified and sent to be quantified.

Quantitation of TMT labelled peptides was performed with PEAKS Studio X**+** search engine, selected ‘Reporter Ion Quantification iTRAQ/TMT’ under the ‘Quantifications’ options. We use Auto‐normalization mode that calculates a global ratio from the total intensity of all labels in all quantifiable peptides. The ‐10LgP, Quality (14) and Reporter Ion Intensity (2.5e4) were used for Spectrum filter and Significance (20 and PEAKSQ method) was used for peptide and protein abundance calculation. For protein quantification, we considered protein groups for peptide uniqueness, used only unique peptides for protein quantification and excluded modified peptides.

#### 2.5.5 Biochemical analyses

Cells or EV samples were lysed with TBS + 1% Triton X‐100 containing proteases inhibitors (Roche) at 4°C for 30 min. Lysates were boiled in non‐reducing Laemmli buffer at 96°C for 5 min and 40 μl of lysates were loaded in 10% Polyacrylamide SDS‐page gels. After electrotransference with a Transfer‐Blot Turbo system (BioRad), membranes were blocked with 5% skimmed‐milk in TBS, 0.1% Tween‐20 for 20 min. EV samples were directly spotted on a nitrocellulose membrane. Immunoblots were revealed with Super Signal® West Femto HRP substrate (Thermo Scientific), and images acquired with a LAS 4000 mini system (General Electrics

### 2.6 Mice experimentation

All animals were housed according to institutional guidelines and all experiments were approved by the Spanish ISCIII Ethical Committee. The experiments were performed in accordance to the guidelines stated in The International Guiding Principles for Biomedical Research involving Animals.

#### 2.6.1 Haematological and bleeding studies

A total of 30 μg of tetraspanin peptides or vehicle solution were subcutaneously injected in the mouse flank twice a week for three weeks. Haematological follow‐up was carried once a week from mice blood samples using an automated hematologic analyser (Diatron^R^). Prior to sacrifice a tail‐bleeding assay was performed using the immersion method (Saito et al., 2016). Briefly, a 5 mm tail amputation was performed and the tail was immersed into 50 ml Falcon tubes containing an isotonic solution at 37°C. The bleeding and rebleeding episodes were quantified for 10 min. The mice were anesthetized with isoflurane and placed on a heating pad in order to maintain the body temperature.

#### 2.6.2 Xenograft and metastasis studies

5‐6‐week‐old female mice (Hsd: Athymic Nude‐Foxn1nu, Jackson laboratory) were subcutaneously injected in one flank with 2 × 10^5^ SK‐MEL‐147 cells. Melanoma cells had been previously pre‐treated twice (days ‐7, ‐3 and 0) with cytopermeable peptides to CD9 or CD63 (2,5 μM) or PBS. Cells were detached with trypsin and resuspended in 100 μl of a 1:1 v/v mixture of cold DMEM:Matrigel and kept on ice until injection. A total of 30 μg of peptides were weekly injected intra‐tumour in 40 μl of PBS. Tumour measurements were performed twice a week with a calliper and tumour volumes were calculated using the standard formula 0.52 x L x W2, in which L is the longest diameter and W is the shortest diameter. After 28 or 35 days, primary tumours were resected after anesthetizing the animals with isoflurane on a heating pad. Three weeks later, mice were sacrificed and lungs were collected for the analysis of metastases.

#### 2.6.3 Pre‐metastatic niche assay

A total of 10 μg of EVs derived from conditioned media of SK‐MEL‐147 cells, treated or not with cytopermeable peptides, were retro‐orbital injected in 5‐6‐week‐old female mice (Hsd: Athymic Nude‐Foxn1nu, Jackson laboratory) twice a week for 3 weeks, followed by intravenous injection of 1 × 10^5^ untreated tumour cells. Mice were sacrificed after 28 days and tissues were dissected for metastasis histopathological analyses.

#### 2.6.4 Histopathological analyses

Primary tumour and selected tissues were washed in fresh PBS and fixed in formalin at room temperature for 24 h. Formalin was eliminated by successive washing with PBS. Fixed samples were paraffin‐embedded, stained with conventional haematoxylin/eosin or stained with the indicated markers and evaluated by bright‐field microscopy.

### 2.7 Confocal fluorescence microscopy

Cells were plated on fibronectin 5 μg/ml (Sigma); fixed with 4% PFA (Electron Microscopy Sciences) for 15 min and, when required, permeabilized using 0.2% Triton X‐100 diluted in TBS. Samples were blocked with TNB at 37°C for 1 h. The corresponding primary antibodies were added, and after extensive washing, the secondary antibodies coupled to Alexa fluorochromes. Incubation with the primary and secondary antibodies were done in the presence of saponin in permeabilized samples. Samples were mounted with Fluoromont‐G (Southern Biotech) to which DAPI (1 μg/ml) had previously been added.

To monitor autophagy flow, we used the LC3‐mTagRFP‐mWasabi‐LC3 construct. Cells were transfected by electroporation and plated on 5 μg/ml FN coated coverslips under normal culture conditions (DMEM 10% FBS) or different fasting times (removing serum from the culture medium). Following these treatments, samples were fixed with 4% PFA and analysed by confocal microscopy.

For mitophagy analysis with mt‐mKeima, cells were transfected by electroporation and 24 h later, images of living cells were obtained by confocal microscopy at 37°C 5% CO_2_. The ratio between the signal intensity at 620 nm obtained by exciting with the 561 laser and the 488 laser was calculated, being this index higher if mitophagy is active in the cell.

Images were acquired with a Leica TCS‐SP5 confocal microscope equipped with Ar and He/Ne lasers, coupled to a Leica DMIRBE inverted epifluorescence microscope (Leica Microsystems, Heidelberg, Germany). Image analyses were done with Image J (NIH) software.

### 2.8 Electron microscopy

Cells were directly fixed on the culture plate with a mixture of 4% paraformaldehyde and 2% glutaraldehyde in 0.1 M phosphate buffer pH 7.4 for 1–2 h at room temperature. After washing with 0.1 M phosphate buffer pH 7.4, samples were incubated for 1 h at 4°C with 1% Osmium Tetroxide in bidistilled water and 1% potassium ferricyanide. Thereafter, samples were treated for one minute with 0.15% Tannic acid in 0.1 M phosphate pH 7.4 and with 2% Uranyl acetate in water for 1 h at room temperature in the dark. The samples were dehydrated with 50%, 75%, 90%, 95% and finally 100% ethanol, in all cases for 10 min at 4°C. Infiltration was done sequentially in epon: ethanol (1: 2), epon: ethanol (1: 1), epon: ethanol (2: 1), for 60 min at room temperature and in 100% epon overnight at 4°C. After this step, the plate was covered with complete epon which was allowed to polymerize for 48 h at 60°C. Epoxy resin, TAAB 812 (TAAB laboratories, Berkshire, England) was used. Ultrafine cuts of 60–70 nm were made on a LEICA ULTRACUT UCT ultramicrotome (Leica, Vienna). The sections were stained with 2% uranyl acetate in water for 7 min and with Reynolds lead citrate for 2 min. For enumeration of intracellular compartments early endosomes were defined as empty endosomal structures, MVBs as endosomal structures with intraluminal vesicles and lysosomes as those endosomal structures showing electrodense material or membrane spirals. EV samples obtained by SEC from cell‐conditioned media were adsorbed on carbon‐coated nickel grids by floating an ionized grid onto a drop of the sample. The grids were contrasted with 2% uranyl acetate. Samples were visualized in a Jeol JEM‐1010 (Jeol, Japan) at 80Kv and images were acquired with a 4KI 4K CMOS camera, F416 from TVIPS (Gauting, Germany). Analysis of the images was performed by using TEM Exosome Analyzer software (Kotrbová et al., 2019).

### 2.9 Flow cytometry

Cells were trypsinized and washed with PBS and fixed with 4% PFA during 10 min at RT. Fixation was neutralized with TBS and then cells were incubated with a blocking solution (5% BSA, 0.1% Saponin, in PBS) during 30 min at RT. Cells were pelleted and resuspended in the Permeabilization solution (PS) (15 mM Glycine, 0.1% Saponin, 10 mM HEPES, 0.5% BSA in PBS) and incubated with the primary antibody of interest diluted in PS for 30 minutes at 4°C and after wash with PBS, with the appropriated secondary antibodies (ThermoFisher Scientific) for 30 minutes at 4°C. Cells incubated with only the secondary antibody were used as a negative control. Lysosomal labelling with Lysotracker Green (Invitrogen) was done following the manufacturer's instructions with a concentration of 75 nM in serum‐free culture medium for 10 minutes at 37°C. After washing with PBS samples were analysed by fluorescence microscopy or flow cytometry. For mitochondria staining and quantitation Mitotracker‐green labelling was carried out following manufacturer´s instructions. 100 nM Mitotracker‐green (Invitrogen) in serum‐free medium was added to the cell culture for 1 h at 37°C before washing with PBS, trispsinization and resuspension in PBS for flow cytometry. Acquisition was performed in either a FACSCalibur or FACSCanto (Becton Dickinson) equipment.

### 2.10 Mitochondrial activity analyses

#### 2.10.1 Isolation of cell membranes

Samples were homogenized using a Teflon‐glass grinder (Heidolph RZR 2020) in 20 volumes of homogenization buffer (1 mM EGTA, 3 mM MgCl_2_, and 50 mMTris‐HCl, pH 7.4) supplemented with 250 mM sucrose. The crude homogenate was subjected to a 1000 g centrifugation for 8 min, and the resultant supernatant was centrifuged again at 18000 g for 15 min (4°C, Microfuge 22R centrifuge, Beckman Coulter). The pellet was washed in 20 volumes of homogenization buffer using an Ultra‐Turrax (T10 basic, IKA) and re‐centrifuged under the same conditions. The homogenate aliquots were stored at ‐80°C until usage. Protein concentration was measured by the Bradford method and adjusted to the required concentrations.

#### 2.10.2 Cell membrane microarray development

Membrane homogenates were resuspended in buffer and printed (4 nL per spot, 3–5 replicates per sample) onto glass slides using a non‐contact microarrayer (Nano_plotter NP 2.1). Microarrays were stored at ‐20°C until usage.

#### 2.10.3 NADH dehydrogenase activity

The standard assay medium for measurement of NADH consumption by NADH dehydrogenase activity consisted of 100 μg/ml cellular membranes homogenates in 50 mM phosphate buffer (pH 7.5). The reaction was started by the addition of 700 μM NADH to the assay medium in absence and presence of 50 μM decylubiquinone (dUQ). The change in absorbance was measured at 340 nm on Scan it for Multiscan spectrophotometer (Thermo Scientific). Control experiments were performed to exclude any interference or substrate interaction.

#### 2.10.4 Succinate dehydrogenase activity

The superoxide production evoked by the activity of the mitochondrial complex II, Succinate dehydrogenase. The assay buffer consisted of a 50 mM phosphate buffer (Na_2_HPO_4_, NaH_2_PO_4_, pH 7.4), 0.5 mg/ml nitro blue tetrazolium (NBT) as a redox dye and 1 mM succinate as a complex II substrate. The reaction was started by the addition of 0.1 mg/ml membranes homogenates for 360 min (25°C) with or without 50 μM dUQ. The NBT reduction evoked by the superoxide generation mediated by CII activity was measured every 5 min spectrophotometrically at 595 nm in a Multiskan microtiter plate reader (Thermo Scientific).

#### 2.10.5 Dihydroorotate dehydrogenase activity

The dihydroorotate dehydrogenase activity assay protocol was similar to that used for succinate dehydrogenase activity using 10 mM dihydroorotate (DOA) instead of succinate. The NBT reduction evoked by the superoxide generation mediated by dihydroorotate dehydrogenase was measured every 5 min spectrophotometrically at 595 nm in a Multiskan microtiter plate reader (Thermo Scientific).

#### 2.10.6 Glycerol 3‐phosphate dehydrogenase activity

The NBT reduction evoked by the superoxide generation mediated by glycerol 3‐phosphate dehydrogenase was measured using the succinate dehydrogenase assay medium with 10 mM glycerol 3‐phosphate (G3P) instead of succinate. Measures were acquired every 5 min spectrophotometrically at 595 nm in a Multiskan microtiter plate reader (Thermo Scientific).

#### 2.10.7 Cytochrome c oxidase assay

The assay buffer for the determination of cytochrome c oxidase activity consisted of a 100 mM phosphate buffer (Na_2_HPO_4_, NaH2PO_4_, pH 7.4), 1.4 mM 3,3′‐diaminobenzidine (DAB) in the absence or in the presence of DOA and G3P (PCT/EP2018/074769). The reaction was started by adding 0.1 mg/ml membranes homogenates to the assay buffer (25°C). DAB oxidation was measured every 5–10 min spectrophotometrically at 460 nm in a Multiskan microtiter plate reader (Thermo Scientific) for 960 min.

#### 2.10.8 Image processing

A Leica DM500 microscope and Leica ICC50 HD camera were coupled to the capturing software LAS EZ 2.0.0. Microphotographs were captured with the same light parameters and the final image was assembled with the software Adobe Photoshop CS5 (Adobe Systems Incorporated, California, USA). Microarrays were also scanned using a scanner (Epson Perfection V750 Pro) and quantified with the MAPIX software (version 7.3.1).

#### 2.10.9 Statistical analyses of mitochondrial ETC activity

Data handling and analysis was carried out using Excel and GraphPad software (version 6.0). Results were expressed as means of independent data points ± S.E.M. In sake of clarity, biochemical data are presented as percentage of controls with or without the application of ETC inhibitors. Data were analysed using paired Student's t‐test, one‐way (followed by Tukey's post hoc test) or two‐way ANOVA (followed by Bonferroni's post hoc test), as appropriate.

### 2.11 MALDI‐MS lipidomic assay

Arrays were covered with a suitable matrix with the aid of a standard glass sublimator (Ace Glass 8233), producing a uniform film of aprox. 0.2 mg/cm^2^. As sublimation is a dry method, it prevents lipid delocalization. 1,5‐diaminonaphtalene was used for negative‐ion mode. Once introduced in the spectrometer, the arrays were scanned as in a MALDI‐imaging experiment. The area of the array was explored following a grid of coordinates separated 150 μm. As the spot of the arrays had a diameter of 450 μm, nine pixels were recorded at each spot. The mass spectrometer used in this work was an LTQ‐Orbitrap XL (Thermo Scientific, San José, CA, USA), equipped with a MALDI source with a N_2_ laser (60 Hz, 100 μJ/pulse maximum power output). The laser spot is an ellipsoid of aprox. 50–60 μm × 140–160 μm. Thus, the size of the spots in the array were chosen as to avoid laser shot overlapping. Two microscans of ten shots at 25μJ of laser energy were used to produce the spectrum of each pixel. Mass resolution was set to 60000 at m/z = 400 in an 550–2000 observation window, in negative‐ion modes.

The spectra were analysed as in a MALDI‐imaging experiment: the whole set of data were loaded as a single experiment. Therefore, data loading included spectra normalization by total ion current, spectra alignment and peak picking, filtering all the m/z with intensity < 0.5 % of the strongest peak in the spectrum. Statistical analysis was carried out using an in‐house made statistical algorithm, based on rank compete (see below) and built in Matlab (MathWorks, Natick, USA), for segmentation, Excel and Unscrambler 9.7 (Camo Analytics, Oslo, Norway), for Principal components analysis.

#### 2.11.1 Lipidomic statistical analysis

Segmentation of the pixels in the image of the array was done using a segmentation algorithm, *RankCompete*, based on the properties of *Markov Chains* to define *Random Walker* competing to divide the imaging experiment into two segments. Using this algorithm as splitting algorithm in a modification of a *Hierarchical Divisive Analysis*, a reliable and robust segmentation of the data was achieved. By definition, the *RankCompet*e algorithm divides the experiment into two segments, however, the data from the array may contain a variable number of segments. Therefore, we used a variation of the Divisive Analysis algorithm (DIANA) to create a variable number of walkers. Thus, the final software is a segmentation algorithm based on DIANA and using *RankCompete* as split function. Once the segments were obtained, the correlation between them was obtained and the value was used to assign a colour to each segment using a colour scale and 1‐correlation between the segments. In this way, the two segments that present the lowest correlation occupy the two extremes of the scale and those segments with more similar average spectra receive colours that are closer in the scale.

## 3 RESULTS

### 3.1 Tetraspanin C‐term modelling

**FIGURE 1:**
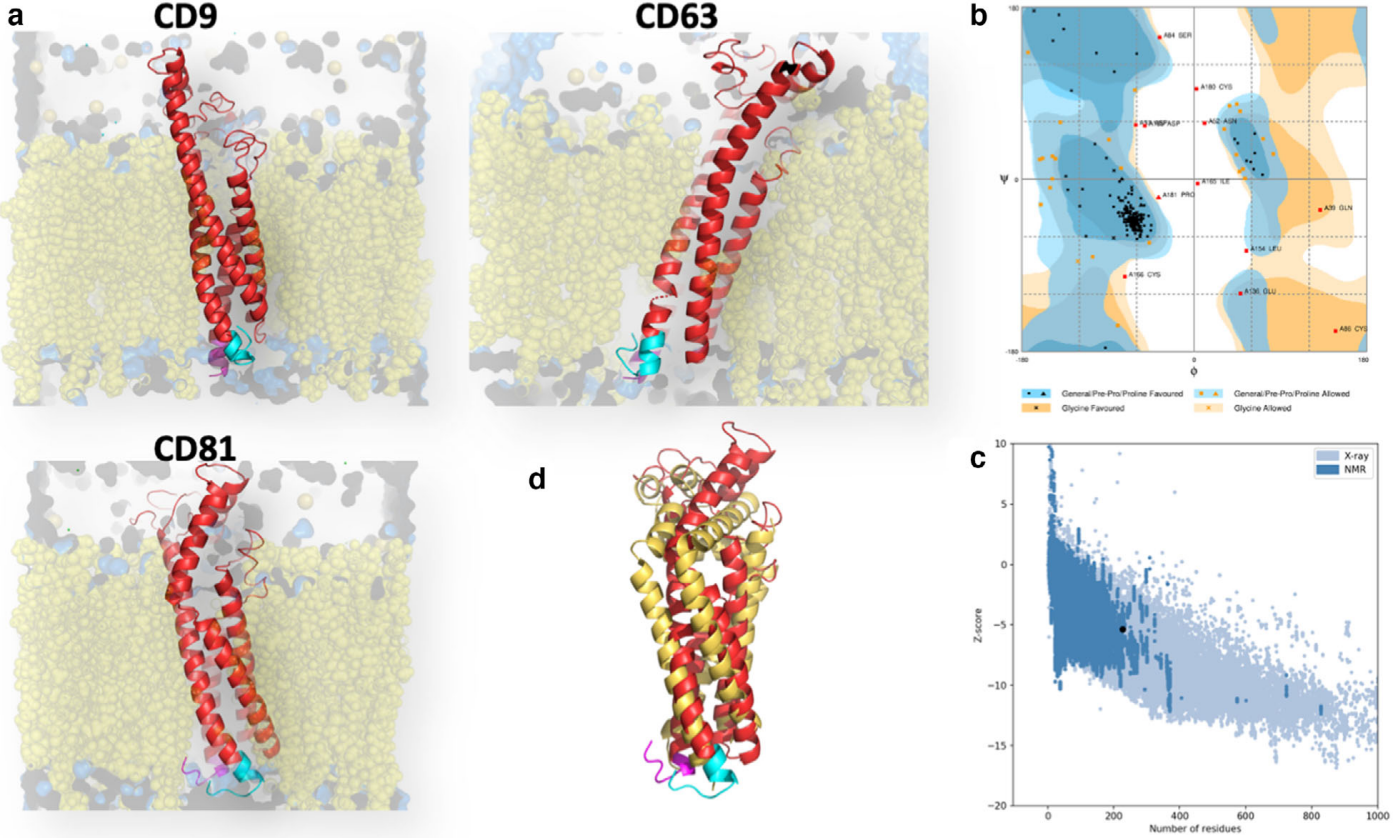
Ab initio modelling of tetraspanins CD9, CD63 and CD81. a. Three‐dimensional structure of tetraspanins CD9, CD63 and CD81 (red, N‐term sequences depicted in magenta and C‐term sequences in blue) inserted in a lipid bilayer (yellow) derived from ab initio calculations as detail under Methods. b. Ramachandran plot of the calculated structure for tetraspanin CD9. c. Validation of the ab initio structure by Protein Software Analysis (ProSA) for CD9. d. Superposition of the ab initio structure of CD81 (red) with the one corresponding to the crystal with the open conformation (yellow) (Zimmerman et al., 2016)

Only the tetraspanin CD81 (Zimmerman et al., 2016) and, during the course of revision of this manuscript, CD9 (Oosterheert et al., 2020; Umeda et al., 2020) and CD53 (Yang et al., 2020) have been so far crystalized. In none of those reports was the structure of the C‐terminal sequence reported in detail; it was too flexible to be determined or even had to be mutated to prevent disulfide crosslinking during purification (Zimmerman et al., 2016). Thus, to gain some insight on the functional effect of the cell‐penetrating peptides based on the C‐terminal region of tetraspanins, we decided to model ab initio the most relevant EV tetraspanins, CD9, CD63 and CD81. We first modelled short sequences using QUARK Software, that uses an atomistic coupling with molecular dynamics replacement method (Xu & Zhang, 2013), and to assemble the fragments we employed the extended C‐QUARK version, which integrates Deep‐Learning strategies (Zhang et al., 2018), further refined with ModRefine software. To produce a protein‐lipid membrane system we employed CHARMM‐GUI with 60 % LPC, 30% POPC and 10% endosomal lipids for lipid composition and a solution containing Na^+^ and Cl^−^ ions to solvate the interior and exterior surfaces (Wu et al., 2014) (Figure 1a). The thickness of the membrane was adjusted by OPM PPSERVER (Lomize et al., 2012). All resulting structures had a Tm‐SCORE ≥ 0.5; were validated by Ramachandran plots (RAMPAGE Tool) (Lovell et al., 2003) (Figure 1b and data not shown, with only 5.3% of residues in the outlier region for CD9; 5.1% for CD63 and 8.5% for CD81), and validated by Protein Software Analysis (ProSA) (Wiederstein & Sippl, 2007) (Figure 1c and data not shown, providing Z scores of ‐5.37 for CD9; ‐5.1 for CD63 and ‐4.72 for CD81, all within the normal parameters for proteins with similar size). We confirmed that the predicted structure for CD81 was overall similar to that of the crystalized molecule (Zimmerman et al., 2016) (Figure 1d).

**FIGURE 2:**
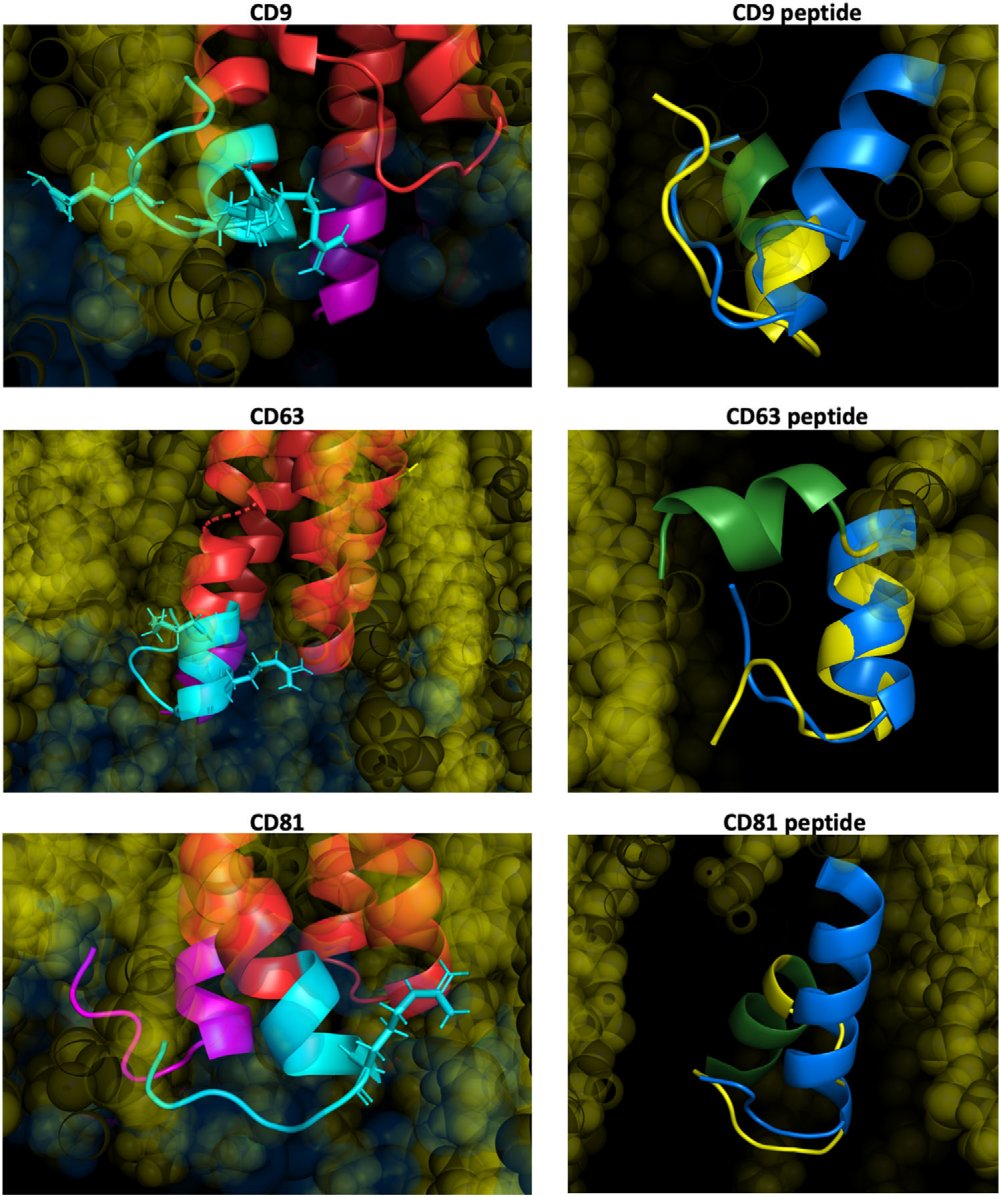
Modelling of cytopermeable peptides. Left panels: detail of the cytosolic regions of tetraspanins CD9, CD63 and CD81 from the ab initio structures. N‐term sequences are depicted in magenta and C‐term sequences in cyan blue. Basic residues are shown in the structure with expanded radical structure. Right panels: Superposition of the modelling of cytopermeable peptides with the tetraspanin C‐terminal region (in blue). The arginine stretch in the peptides is depicted in green and the sequence corresponding to the C‐terminal region in yellow

Surprisingly, the C‐terminal sequence of all three tetraspanins presented a bent conformation (Figure 2 left panels) in which basic residues interacted with the cytosolic surface of the lipid bilayer imposing a loop‐like structure. Modelling of the cell‐penetrating guanidinium‐rich peptides, carrying the C‐terminal sequences of the selected tetraspanins, confirmed that Arginine homo‐oligomers formed fork‐like interactions with the cytosolic surface of the lipid bilayer (Stanzl et al., 2013) thus remaining attached to the membrane and exposing the sequence corresponding to the C‐terminal region in a similar structure to this same region in the full tetraspanin molecule (Figure 2 right panels). These structural data would support that the cytopermeable peptides block tetraspanin function by competing with tetraspanin interactors at this C‐terminal loop‐like structure.

### 3.2 CD9 regulates early endosome formation and EV composition

Both CD9 and CD63 are important regulators of melanoma progression and metastasis (Fan et al., 2010; Kondoh et al., 1993; Si & Hersey, 1993), so we decided to use a human melanoma cell line for our studies. Our previous analyses employing CD81 cell‐penetrating peptide revealed that these peptides have only a functional effect on cells expressing the targeted tetraspanins (Martínez Del Hoyo et al., 2015), so we first screened different melanoma cell lines for their expression of tetraspanins and their secretion in extracellular vesicles. Both CD63 and CD9 were markedly downregulated in highly metastatic cell lines, so for all subsequent experiments we chose SK‐MEL‐147 cells, with appreciable metastatic ability but detectable expression of both CD9 and CD63 tetraspanins.

**FIGURE 3:**
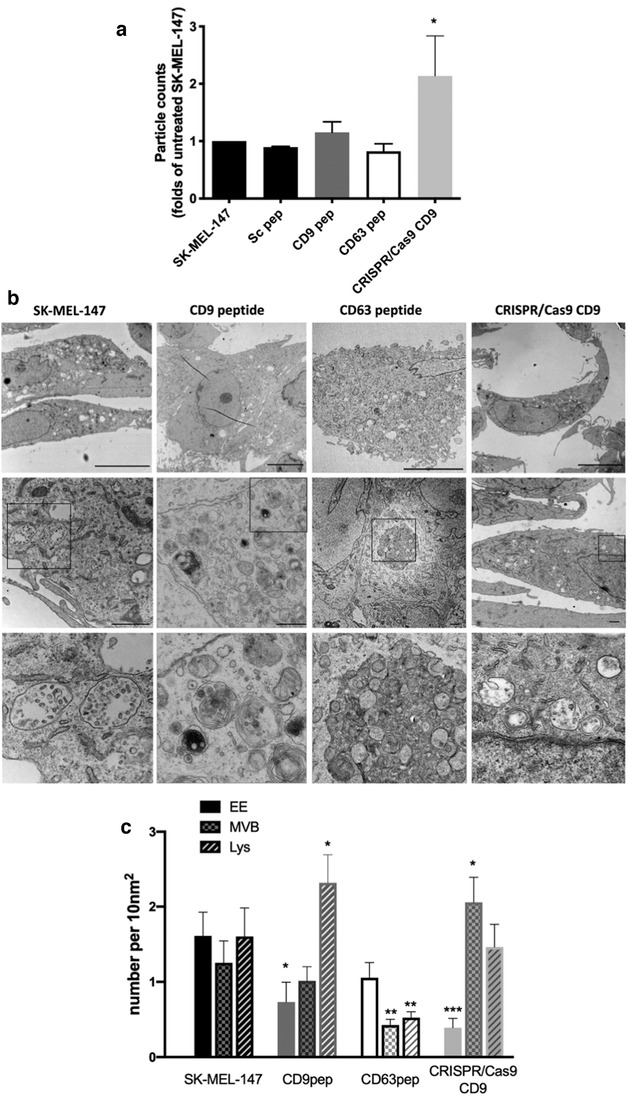
Effect of CD9 cytopermeable peptide or gene deletion on EV secretion and the endolysosomal compartments. a. NTA analyses of the number of particles detected in 7d conditioned media as folds of untreated sample in each independent experimental replicate, performed by duplicated. Data are represented as the mean ± SEM of a minimum of five independent measurements. * *P* < 0.05 in a Student T‐test b. Representative electron micrographs of untreated SK‐MEL‐147 cells and cells treated for 7d with CD9 or CD63 peptides or untreated CD9 KO cells. Bottom images correspond to higher magnification of the field denoted by a square in middle images depicting the most representative subcellular compartments in each condition. MVBs are shown in wt and CD9 KO samples. Late endosomes and lysosomes (electrodense or multimembrane structures) are abundant in CD9 peptide‐treated cells, while an autophagic structure is observed in the sample treated with CD63 peptide. Bars = 10 μm (upper panels), 1 μm in middle panels. c. Number of early endosomes, MVBs and lysosomes was quantitated on EM images in at least 10 cells/condition. Data are represented as the mean ± SEM. * *P* < 0.05 ** *P* < 0.01 *** *P* < 0.001 in a Mann‐Whitney test

EV secretion is a long and complex phenomenon, starting with the internalization of plasma membrane molecules, endosome maturation and fusion of multivesicular bodies with the plasma membrane. Therefore, we decided to make repeated treatments to melanoma cells during a 7d period with peptides of CD9, a scrambled control or CD63 as a specificity control. In addition, we generated CD9‐KO cells using the CRISPR/Cas9 system (Supplementary Figure 1). Conditioned media was collected and particles directly counted by NTA. Treatment of cells with the tetraspanin‐targeted peptides didn´t significantly affect total EV numbers, although a mild reduction was observed in the conditioned media of cells treated with CD63 peptide, while CD9‐peptide treatment induced slight increases in EV number. In contrast, the number of EVs secreted into conditioned media was significantly increased upon CD9 gene deletion (Figure 3a). Few particles were measured in non‐conditioned medium with peptide (Supplementary Figure 2, note the logarithmic range on the scale).

To understand these changes, we analysed in detail the subcellular ultrastructure of the different melanoma cell cultures. Both CD9 KO cells and those treated with the CD9 peptide, showed a reduction in the number of early endosomes, which appeared as clear vesicular structures in electron microscopy, even at low magnification (Figure 3b upper panels and quantitated in Figure 3c), and after EEA1 staining by confocal microscopy (Supplementary Figure 5A). However, while in CD9 peptide‐treated cells there was a greater prevalence of lysosomes, which could be observed by EM as electrodense or multimembranous structures; in CD9 KO cells the increase was detected in the MVB compartment (Figure 3b and c). A similar profile was observed when markers for early endosomes (EEA‐1), MVBs (Hrs) or lysosomes (LAMP‐1) were analysed by flow cytometry in permeabilized samples (Supplementary Figure 5C). Consistent with the described effect of CD63 gene deletion (Hurwitz et al., 2016), CD63 peptide treatment induced a reduction in late endosomal compartments and the generation of large autophagosome structures in many cells (Figure 3b).

This differential effect of CD9 peptide and KO cells in late endosomal compartment distribution, could be explained by the compensatory increase of other tetraspanins in CD9 KO cells. We know from our previous experience in working with tetraspanin silencing and gene deletion, that other tetraspanins expression is commonly upregulated (Barreiro et al., 2005), probably as a compensatory mechanism. Therefore, we usually work only with the first cellular passages after gene deletion. However, in this melanoma cell line, compensatory upregulation of CD63 was already observed in these early passages, both by flow cytometry and Western‐blot (Supplementary Figure 1C and D). The expression levels of CD81 at the plasma membrane remain rather unaffected, as measured by flow cytometry in non‐permeabilized samples (not shown), but were slightly diminished when overall expression was analysed in whole cell lysates or by flow cytometry in permeabilized samples (Supplementary Figure 1C and D). No significant changes in overall tetraspanin expression were detected upon treatment with cytopermeable peptides.

Intriguingly, even though particle counts on conditioned media form CD9 KO cells were increased, both when measured without any isolation procedure or after SEC purification, and assessed by either NTA or electron microscopy analyses (Supplementary Figures 2–4), the SEC‐isolated EV samples from CD9 KO cells showed a reduced total protein content, as compared to samples obtained from conditioned media from the same number of untreated wt SK‐MEL‐147 cells or from cells treated with the different peptides, except for CD9 peptide, that showed a similar trend (Supplementary Figure 4B), suggesting that the total protein load per vesicle is reduced in these samples.

**FIGURE 4:**
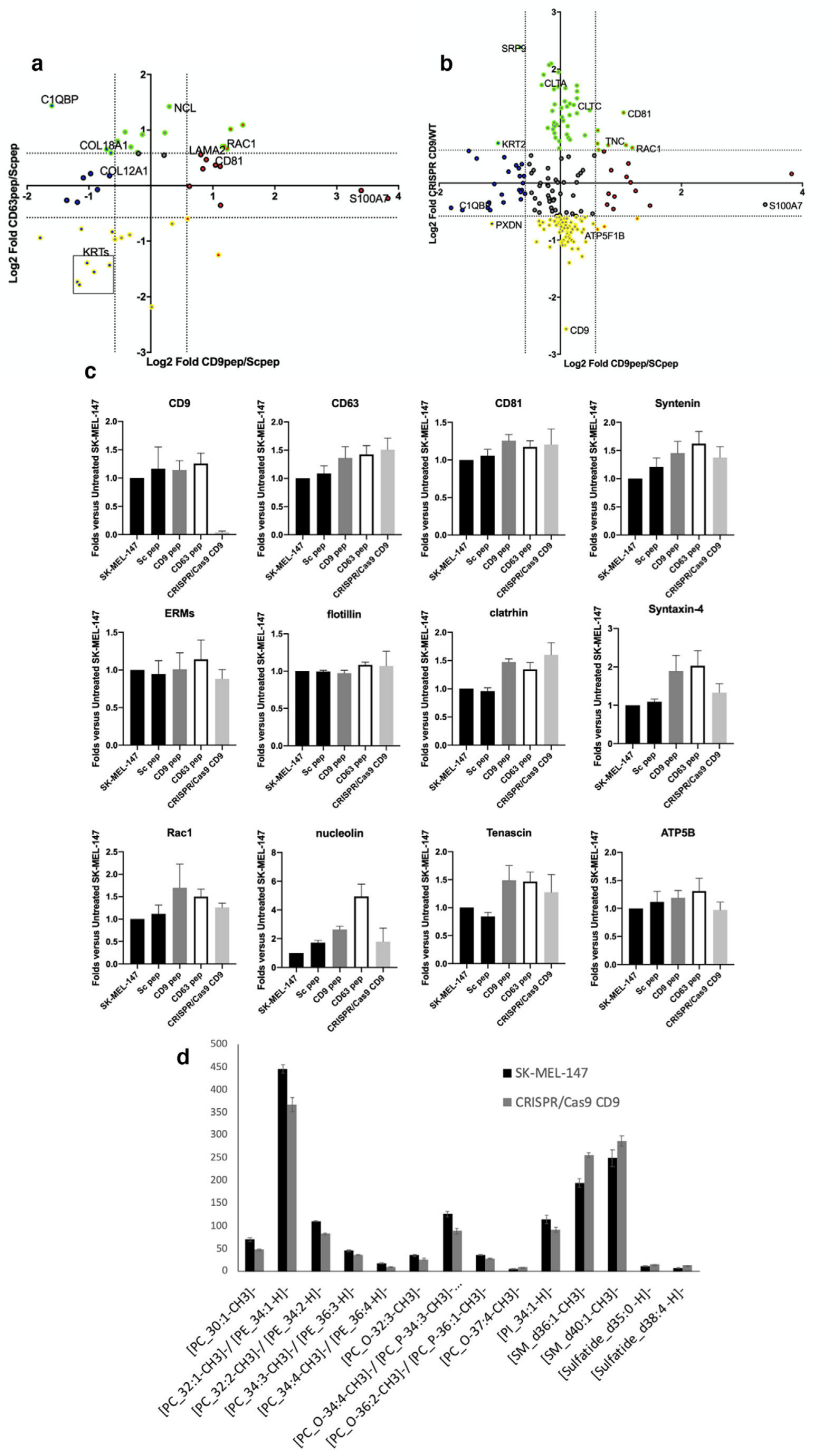
Effect of tetraspanin cytopermeable peptides and CD9 gene deletion on EV composition. a. Two‐dimensional plot comparing those proteins that showed differences in their sorting after treatment of SK‐MEL‐147 cells with CD63 or CD9 cytopermeable peptides, or b. after treatment of SK‐MEL‐147 cells with CD9 cytopermeable peptide or complete gene deletion. Only statistically significant proteins are plotted (Log_10_[P]≥2). Dotted lines in both axis mark ± 0.58, which represents 1.5 and 0.66 times Fold change thresholds (Log_2_[1.5] = 0.58, Log_2_[0.66] = ‐0.58). Some of the featured proteins are marked in the plot. c. Quantitative analyses of dot‐blots from lysates of SEC isolated EV samples revealed with specific antibodies against the indicated proteins. Data correspond to the mean ± SEM of three independent experiments. d. MALDI‐MS lipidomic assays of EV samples were performed on membrane arrays from two independent replicates of SEC‐isolated EV samples analysed by quadruplicated. Data are represented as the mean ± SEM. Only those lipid forms that showed statistically significant differences in both experiments are represented. In all cases *P* < 0.05 in a Student T‐test

The composition of SEC isolated EVs was determined by proteomic iTRAQ, unveiling composition changes (Figure 4a, b and Supplementary Tables 1 and 2). In these analyses we were able to detect and analyse 1093 proteins with at least two unique peptides. Some of the changes observed were further validated by biochemical methods on EV lysates after SEC purification (Figure 4c). CD9 deletion or treatment of cells with CD9 peptide induced an increment in CD81 on EVs, which was not detected with CD63 peptide treatment (Figure 4c and Table 1). All three conditions showed a slight increase in CD63 or Syntenin‐1 recovery on EVs, while other EV abundant proteins such as flotillin or ERMs were not affected (Table 1, 2 and Figure 4c). Other markers of sEVs were also clearly overrepresented in CD9KO cells, such as Syndecan 1 and 4, as well as different components of the clathrin complex (Table 2 and Figures 4b and 4c). Clathrin differences were not significant in CD9 peptide treated samples in proteomic analyses but could also be detected in biochemical studies (Figure 4b and c). CD9‐peptide treated samples also showed increased presence of laminin and S100‐A7 calcium binding protein (Figure 4a, b and Table 1). Syntaxin‐4 was upregulated in both peptide‐treated samples, while other changes were more specific of CD63 peptide treatment, such as the upregulation of nucleolin (Figure 4a and c). A bigger overlap was found in the repertoire of proteins downregulated in both peptide treatments (Figure 4a and Table 1), which included some members of the keratin family, which have been previously identified in a subpopulation of small EVs (sEVs) (Kowal et al., 2016). CD9‐peptide treated samples showed downregulation of other markers found in that same subpopulation, such as collagen or complement components. Another extracellular matrix molecule, tenascin, which has recently been reported to be secreted via caveolin‐dependent EVs (Albacete‐Albacete et al., 2020), was upregulated in both peptides and CD9KO cells (Tables 1 and 2 and Figure 4b and c) as well as the small GTPase Rac1. Proteomic data were analysed using STRING, which revealed the presence of some protein clades in the proteins that were altered in EV samples. GO analyses revealed that many are related to cell‐adhesion or extracellular matrix (in red in Supplementary Figure 6D and E) or to vesicle transport (highlighted in dark blue).

**TABLE 1:** List of proteins found to be upregulated more than 1.5 times (pink) or downregulated below 0.66 (green) in EVs isolated from conditioned media of SK‐MEL‐147 cells treated with either CD9 or CD63 peptides, related to samples from scramble peptide treated cells

Gene Name	Accession	Significance	Ratio CD9vpep/Sc pep	Ratio CD63 pep/Sc pep	Description
SRPX	P78539	112.75	0.29	0.52	Sushi repeat‐containing protein SRPX OS = Homo sapiens OX = 9606 GN = SRPX PE = 1 SV = 1
C1QBP	Q07021	200	0.33	2.71	Complement component 1 Q subcomponent‐binding protein mitochondrial OS = Homo sapiens OX = 9606 GN = C1QBP PE = 1 SV = 1
MDK	P21741	40.04	0.39	0.83	Midkine OS = Homo sapiens OX = 9606 GN = MDK PE = 1 SV = 1
KRT1	P04264	90.94	0.44	0.3	Keratin type II cytoskeletal 1 OS = Homo sapiens OX = 9606 GN = KRT1 PE = 1 SV = 6
THBS1	P07996	48.85	0.44	0.81	Thrombospondin‐1 OS = Homo sapiens OX = 9606 GN = THBS1 PE = 1 SV = 2
KRT9	P35527	108.42	0.45	0.29	Keratin type I cytoskeletal 9 OS = Homo sapiens OX = 9606 GN = KRT9 PE = 1 SV = 3
PXDN	Q92626	40.85	0.46	0.58	Peroxidasin homolog OS = Homo sapiens OX = 9606 GN = PXDN PE = 1 SV = 2
RPL27	P61353	33.08	0.47	1.1	60S ribosomal protein L27 OS = Homo sapiens OX = 9606 GN = RPL27 PE = 1 SV = 2
KRT2	P35908	66.84	0.49	0.38	Keratin type II cytoskeletal 2 epidermal OS = Homo sapiens OX = 9606 GN = KRT2 PE = 1 SV = 2
RPL34	P49207	22.77	0.51	1.16	60S ribosomal protein L34 OS = Homo sapiens OX = 9606 GN = RPL34 PE = 1 SV = 3
KRT10	P13645	104.91	0.53	0.34	Keratin type I cytoskeletal 10 OS = Homo sapiens OX = 9606 GN = KRT10 PE = 1 SV = 6
THBS2	P35442	23.24	0.55	0.95	Thrombospondin‐2 OS = Homo sapiens OX = 9606 GN = THBS2 PE = 1 SV = 2
COL18A1	P39060	111.95	0.61	1.57	Collagen alpha‐1(XVIII) chain OS = Homo sapiens OX = 9606 GN = COL18A1 PE = 1 SV = 5
KRT5	P13647	44.17	0.63	0.37	Keratin type II cytoskeletal 5 OS = Homo sapiens OX = 9606 GN = KRT5 PE = 1 SV = 3
COL12A1	Q99715	22.05	0.63	1.13	Collagen alpha‐1(XII) chain OS = Homo sapiens OX = 9606 GN = COL12A1 PE = 1 SV = 2
CCN1	O00622	65.49	0.64	1.5	Protein CYR61 OS = Homo sapiens OX = 9606 GN = CYR61 PE = 1 SV = 1
CHMP2A	O43633	23.5	0.65	0.56	Charged multivesicular body protein 2a OS = Homo sapiens OX = 9606 GN = CHMP2A PE = 1 SV = 1
ANXA2	P07355	40.21	0.67	0.51	Annexin A2 OS = Homo sapiens OX = 9606 GN = ANXA2 PE = 1 SV = 2
GPC1	P35052	39.05	0.69	1.74	Glypican‐1 OS = Homo sapiens OX = 9606 GN = GPC1 PE = 1 SV = 2
KRT16	P08779	21.39	0.72	0.52	Keratin type I cytoskeletal 16 OS = Homo sapiens OX = 9606 GN = KRT16 PE = 1 SV = 4
NPM1	P06748	68	0.75	1.95	Nucleophosmin OS = Homo sapiens OX = 9606 GN = NPM1 PE = 1 SV = 2
EEF1A2	Q05639	20.56	0.79	0.54	Elongation factor 1‐alpha 2 OS = Homo sapiens OX = 9606 GN = EEF1A2 PE = 1 SV = 1
LOXL2	Q9Y4K0	41.24	0.8	1.62	Lysyl oxidase homolog 2 OS = Homo sapiens OX = 9606 GN = LOXL2 PE = 1 SV = 1
HSPG2	P98160	43.16	0.86	1.51	Basement membrane‐specific heparan sulfate proteoglycan core protein OS = Homo sapiens OX = 9606 GN = HSPG2 PE = 1 SV = 4
SDC4	P31431	52.84	0.91	1.89	Syndecan‐4 OS = Homo sapiens OX = 9606 GN = SDC4 PE = 1 SV = 2
H1‐4	P10412	58.18	1.01	0.22	Histone H1.4 OS = Homo sapiens OX = 9606 GN = HIST1H1E PE = 1 SV = 2
SRPX2	O60687	23.31	1.17	1.93	Sushi repeat‐containing protein SRPX2 OS = Homo sapiens OX = 9606 GN = SRPX2 PE = 1 SV = 1
NCL	P19338	50.66	1.23	2.68	Nucleolin OS = Homo sapiens OX = 9606 GN = NCL PE = 1 SV = 3
PSMA1	P25786	23.38	1.27	0.62	Proteasome subunit alpha type‐1 OS = Homo sapiens OX = 9606 GN = PSMA1 PE = 1 SV = 1
LTF	E7ER44	23.1	1.51	0.66	Lactotransferrin OS = Homo sapiens OX = 9606 GN = LTF PE = 1 SV = 1
ALB	P02768	25.98	1.54	0.99	Serum albumin OS = Homo sapiens OX = 9606 GN = ALB PE = 1 SV = 2
LAMA2	P24043	24.73	1.75	1.47	Laminin subunit alpha‐2 OS = Homo sapiens OX = 9606 GN = LAMA2 PE = 1 SV = 4
LGALS3BP	Q08380	22.23	1.79	1.23	Galectin‐3‐binding protein OS = Homo sapiens OX = 9606 GN = LGALS3BP PE = 1 SV = 1
SLC38A2	Q96QD8	24.91	1.86	1.38	Sodium‐coupled neutral amino acid transporter 2 OS = Homo sapiens OX = 9606 GN = SLC38A2 PE = 1 SV = 2
CD81	P60033	27.99	2.06	1.29	CD81 antigen OS = Homo sapiens OX = 9606 GN = CD81 PE = 1 SV = 1
GJC1	P36383	200	2.13	0.42	Gap junction gamma‐1 protein OS = Homo sapiens OX = 9606 GN = GJC1 PE = 1 SV = 2
AHSA1	O95433	23.74	2.17	1.27	Activator of 90 kDa heat shock protein ATPase homolog 1 OS = Homo sapiens OX = 9606 GN = AHSA1 PE = 1 SV = 1
FABP5	Q01469	32.35	2.18	0.78	Fatty acid‐binding protein epidermal OS = Homo sapiens OX = 9606 GN = FABP5 PE = 1 SV = 3
GNB2	P62879	33.59	2.23	1.63	Guanine nucleotide‐binding protein G(I)/G(S)/G(T) subunit beta‐2 OS = Homo sapiens OX = 9606 GN = GNB2 PE = 1 SV = 3
RAC1	P63000	27.6	2.27	1.63	Ras‐related C3 botulinum toxin substrate 1 OS = Homo sapiens OX = 9606 GN = RAC1 PE = 1 SV = 1
HIST1H4A	P62805	47.29	2.35	1.58	Histone H4 OS = Homo sapiens OX = 9606 GN = HIST1H4A PE = 1 SV = 2
TUBB3	Q13509	24.47	2.44	2.02	Tubulin beta‐3 chain OS = Homo sapiens OX = 9606 GN = TUBB3 PE = 1 SV = 2
TUBA1C	Q9BQE3	33.16	2.8	2.13	Tubulin alpha‐1C chain OS = Homo sapiens OX = 9606 GN = TUBA1C PE = 1 SV = 1
S100A7	P31151	57.05	10.53	0.94	Protein S100‐A7 OS = Homo sapiens OX = 9606 GN = S100A7 PE = 1 SV = 4
SERPINB3	P29508	153.52	14.21	0.85	Serpin B3 OS = Homo sapiens OX = 9606 GN = SERPINB3 PE = 1 SV = 2

Only those proteins quantitated with two unique peptides were considered. Spectrum filter and significance (20 and PEAKSQ method) was used

**TABLE 2:** List of proteins found to be upregulated more than 1.5 times (pink) or downregulated below 0.66 (green) in EVs isolated from conditioned media of CD9 KO cells, related to samples from SK‐MEL‐147 cells

Gene Name	Accession	Significance	Ratio CRISPR Cas9 CD9/SK‐MEL‐147	Description
CD9	P21926	110.47	0.17	CD9 antigen OS = Homo sapiens OX = 9606 GN = CD9 PE = 1 SV = 4
PTX3	P26022	42.68	0.38	Pentraxin‐related protein PTX3 OS = Homo sapiens OX = 9606 GN = PTX3 PE = 1 SV = 3
RACK1	P63244	73.58	0.41	Receptor of activated protein C kinase 1 OS = Homo sapiens OX = 9606 GN = RACK1 PE = 1 SV = 3
RAN	P62826	46.74	0.45	GTP‐binding nuclear protein Ran OS = Homo sapiens OX = 9606 GN = RAN PE = 1 SV = 3
PSMB1	P20618	23.97	0.45	Proteasome subunit beta type‐1 OS = Homo sapiens OX = 9606 GN = PSMB1 PE = 1 SV = 2
OGN	P20774	32.54	0.46	Mimecan OS = Homo sapiens OX = 9606 GN = OGN PE = 1 SV = 1
RPSA	P08865	39.82	0.47	40S ribosomal protein SA OS = Homo sapiens OX = 9606 GN = RPSA PE = 1 SV = 4
UXS1	Q8NBZ7	37.9	0.47	UDP‐glucuronic acid decarboxylase 1 OS = Homo sapiens OX = 9606 GN = UXS1 PE = 1 SV = 1
PHGDH	A0A286YF22	31.03	0.48	D‐3‐phosphoglycerate dehydrogenase OS = Homo sapiens OX = 9606 GN = PHGDH PE = 1 SV = 1
HIST1H1E	P10412	62.93	0.49	Histone H1.4 OS = Homo sapiens OX = 9606 GN = HIST1H1E PE = 1 SV = 2
C3	P01024	59.66	0.49	Complement C3 OS = Homo sapiens OX = 9606 GN = C3 PE = 1 SV = 2
ASNS	P08243	31.46	0.49	Asparagine synthetase [glutamine‐hydrolyzing] OS = Homo sapiens OX = 9606 GN = ASNS PE = 1 SV = 4
HBG2	E9PBW4	29.53	0.49	Haemoglobin subunit gamma‐2 OS = Homo sapiens OX = 9606 GN = HBG2 PE = 1 SV = 1
HBG1	P69891	29.53	0.49	Haemoglobin subunit gamma‐1 OS = Homo sapiens OX = 9606 GN = HBG1 PE = 1 SV = 2
PZP	P20742	28.45	0.49	Pregnancy zone protein OS = Homo sapiens OX = 9606 GN = PZP PE = 1 SV = 4
H1‐5	P16401	22.96	0.49	Histone H1.5 OS = Homo sapiens OX = 9606 GN = HIST1H1B PE = 1 SV = 3
HIST1H1C	P16403	40.49	0.5	Histone H1.2 OS = Homo sapiens OX = 9606 GN = HIST1H1C PE = 1 SV = 2
SHMT2	P34897	30.7	0.5	Serine hydroxymethyltransferase mitochondrial OS = Homo sapiens OX = 9606 GN = SHMT2 PE = 1 SV = 3
UBA1	P22314	39.49	0.51	Ubiquitin‐like modifier‐activating enzyme 1 OS = Homo sapiens OX = 9606 GN = UBA1 PE = 1 SV = 3
RBBP7	E9PC52	40.77	0.52	Histone‐binding protein RBBP7 OS = Homo sapiens OX = 9606 GN = RBBP7 PE = 1 SV = 1
HBB	P68871	33.94	0.52	Haemoglobin subunit beta OS = Homo sapiens OX = 9606 GN = HBB PE = 1 SV = 2
VTN	P04004	58.13	0.53	Vitronectin OS = Homo sapiens OX = 9606 GN = VTN PE = 1 SV = 1
PRSS23	O95084	54.26	0.54	Serine protease 23 OS = Homo sapiens OX = 9606 GN = PRSS23 PE = 1 SV = 1
HBA1	P69905	56.28	0.55	Haemoglobin subunit alpha OS = Homo sapiens OX = 9606 GN = HBA1 PE = 1 SV = 2
RPL10A	P62906	42.41	0.55	60S ribosomal protein L10a OS = Homo sapiens OX = 9606 GN = RPL10A PE = 1 SV = 2
PKM	P14618	25.52	0.55	Pyruvate kinase PKM OS = Homo sapiens OX = 9606 GN = PKM PE = 1 SV = 4
CTSC	P53634	22.77	0.55	Dipeptidyl peptidase 1 OS = Homo sapiens OX = 9606 GN = CTSC PE = 1 SV = 2
PGK1	P00558	21.99	0.56	Phosphoglycerate kinase 1 OS = Homo sapiens OX = 9606 GN = PGK1 PE = 1 SV = 3
ATP5F1B	P06576	37.69	0.57	ATP synthase subunit beta mitochondrial OS = Homo sapiens OX = 9606 GN = ATP5B PE = 1 SV = 3
EEF1G	P26641	36.09	0.57	Elongation factor 1‐gamma OS = Homo sapiens OX = 9606 GN = EEF1G PE = 1 SV = 3
FGB	P02675	33.27	0.57	Fibrinogen beta chain OS = Homo sapiens OX = 9606 GN = FGB PE = 1 SV = 2
DHX15	O43143	25.37	0.57	Pre‐mRNA‐splicing factor ATP‐dependent RNA helicase DHX15 OS = Homo sapiens OX = 9606 GN = DHX15 PE = 1 SV = 2
PSMB7	Q99436	20.2	0.57	Proteasome subunit beta type‐7 OS = Homo sapiens OX = 9606 GN = PSMB7 PE = 1 SV = 1
CCT2	P78371	30.82	0.58	T‐complex protein 1 subunit beta OS = Homo sapiens OX = 9606 GN = CCT2 PE = 1 SV = 4
TOR1B	O14657	29.45	0.58	Torsin‐1B OS = Homo sapiens OX = 9606 GN = TOR1B PE = 1 SV = 2
GAPDH	P04406	52.04	0.59	Glyceraldehyde‐3‐phosphate dehydrogenase OS = Homo sapiens OX = 9606 GN = GAPDH PE = 1 SV = 3
RPLP2	P05387	31.41	0.59	60S acidic ribosomal protein P2 OS = Homo sapiens OX = 9606 GN = RPLP2 PE = 1 SV = 1
AHCY	P23526	30.75	0.59	Adenosylhomocysteinase OS = Homo sapiens OX = 9606 GN = AHCY PE = 1 SV = 4
CCT5	P48643	25.56	0.59	T‐complex protein 1 subunit epsilon OS = Homo sapiens OX = 9606 GN = CCT5 PE = 1 SV = 1
COMP	P49747	31.54	0.6	Cartilage oligomeric matrix protein OS = Homo sapiens OX = 9606 GN = COMP PE = 1 SV = 2
PYGL	P06737	29.36	0.6	Glycogen phosphorylase liver form OS = Homo sapiens OX = 9606 GN = PYGL PE = 1 SV = 4
ACOT7	O00154	26.5	0.6	Cytosolic acyl coenzyme A thioester hydrolase OS = Homo sapiens OX = 9606 GN = ACOT7 PE = 1 SV = 3
XPNPEP1	Q9NQW7	23.22	0.6	Xaa‐Pro aminopeptidase 1 OS = Homo sapiens OX = 9606 GN = XPNPEP1 PE = 1 SV = 3
CCT4	P50991	20.33	0.6	T‐complex protein 1 subunit delta OS = Homo sapiens OX = 9606 GN = CCT4 PE = 1 SV = 4
PXDN	Q92626	60.89	0.61	Peroxidasin homolog OS = Homo sapiens OX = 9606 GN = PXDN PE = 1 SV = 2
LUM	P51884	43.89	0.61	Lumican OS = Homo sapiens OX = 9606 GN = LUM PE = 1 SV = 2
EEF2	P13639	20.15	0.61	Elongation factor 2 OS = Homo sapiens OX = 9606 GN = EEF2 PE = 1 SV = 4
RPL28	P46779	35.44	0.62	60S ribosomal protein L28 OS = Homo sapiens OX = 9606 GN = RPL28 PE = 1 SV = 3
LCAT	P04180	26.06	0.62	Phosphatidylcholine‐sterol acyltransferase OS = Homo sapiens OX = 9606 GN = LCAT PE = 1 SV = 1
NAA15	Q9BXJ9	26.03	0.62	N‐alpha‐acetyltransferase 15 NatA auxiliary subunit OS = Homo sapiens OX = 9606 GN = NAA15 PE = 1 SV = 1
CFB	P00751	25.37	0.62	Complement factor B OS = Homo sapiens OX = 9606 GN = CFB PE = 1 SV = 2
RPS3	P23396	23.03	0.62	40S ribosomal protein S3 OS = Homo sapiens OX = 9606 GN = RPS3 PE = 1 SV = 2
RPL18A	M0R117	24.51	0.63	60S ribosomal protein L18a OS = Homo sapiens OX = 9606 GN = RPL18A PE = 1 SV = 1
CCT8	P50990	23	0.63	T‐complex protein 1 subunit theta OS = Homo sapiens OX = 9606 GN = CCT8 PE = 1 SV = 4
ALDH1A3	P47895	21.75	0.63	Aldehyde dehydrogenase family 1 member A3 OS = Homo sapiens OX = 9606 GN = ALDH1A3 PE = 1 SV = 2
RGN	Q15493	40.54	0.64	Regucalcin OS = Homo sapiens OX = 9606 GN = RGN PE = 1 SV = 1
F13A1	P00488	30.72	0.64	Coagulation factor XIII A chain OS = Homo sapiens OX = 9606 GN = F13A1 PE = 1 SV = 4
SERPINF1	P36955	26.48	0.64	Pigment epithelium‐derived factor OS = Homo sapiens OX = 9606 GN = SERPINF1 PE = 1 SV = 4
TUBB3	Q13509	31.77	0.65	Tubulin beta‐3 chain OS = Homo sapiens OX = 9606 GN = TUBB3 PE = 1 SV = 2
AARS1	P49588	20.48	0.65	Alanine–tRNA ligase cytoplasmic OS = Homo sapiens OX = 9606 GN = AARS PE = 1 SV = 2
KARS1	Q15046	20.39	0.65	Lysine–tRNA ligase OS = Homo sapiens OX = 9606 GN = KARS PE = 1 SV = 3
CD63	P08962	11.11	1.49	CD63 antigen OS = Homo sapiens OX = 9606 GN = CD63 PE = 1 SV = 2
GJA1	P17302	23.95	1.53	Gap junction alpha‐1 protein OS = Homo sapiens OX = 9606 GN = GJA1 PE = 1 SV = 2
RAC1	P63000	37.16	1.54	Ras‐related C3 botulinum toxin substrate 1 OS = Homo sapiens OX = 9606 GN = RAC1 PE = 1 SV = 1
GJC1	P36383	200	1.59	Gap junction gamma‐1 protein OS = Homo sapiens OX = 9606 GN = GJC1 PE = 1 SV = 2
TNC	P24821	20.5	1.59	Tenascin OS = Homo sapiens OX = 9606 GN = TNC PE = 1 SV = 3
MFGE8	Q08431	22.13	1.62	Lactadherin OS = Homo sapiens OX = 9606 GN = MFGE8 PE = 1 SV = 2
KRT2	P35908	89.71	1.63	Keratin type II cytoskeletal 2 epidermal OS = Homo sapiens OX = 9606 GN = KRT2 PE = 1 SV = 2
LASP1	Q14847	20.75	1.67	LIM and SH3 domain protein 1 OS = Homo sapiens OX = 9606 GN = LASP1 PE = 1 SV = 2
DNAJA1	P31689	21.64	1.7	DnaJ homolog subfamily A member 1 OS = Homo sapiens OX = 9606 GN = DNAJA1 PE = 1 SV = 2
SDC1	P18827	25.22	1.74	Syndecan‐1 OS = Homo sapiens OX = 9606 GN = SDC1 PE = 1 SV = 3
LRRC15	Q8TF66	21.2	1.74	Leucine‐rich repeat‐containing protein 15 OS = Homo sapiens OX = 9606 GN = LRRC15 PE = 2 SV = 2
WWP2	O00308	21.48	1.79	NEDD4‐like E3 ubiquitin‐protein ligase WWP2 OS = Homo sapiens OX = 9606 GN = WWP2 PE = 1 SV = 2
LDLR	P01130	20.49	1.81	Low‐density lipoprotein receptor OS = Homo sapiens OX = 9606 GN = LDLR PE = 1 SV = 1
GRB2	P62993	24.3	1.84	Growth factor receptor‐bound protein 2 OS = Homo sapiens OX = 9606 GN = GRB2 PE = 1 SV = 1
LTF	E7ER44	41.82	1.9	Lactotransferrin OS = Homo sapiens OX = 9606 GN = LTF PE = 1 SV = 1
PRNP	P04156	29.87	1.93	Major prion protein OS = Homo sapiens OX = 9606 GN = PRNP PE = 1 SV = 1
PROCR	Q9UNN8	36.51	1.96	Endothelial protein C receptor OS = Homo sapiens OX = 9606 GN = PROCR PE = 1 SV = 1
SDC4	P31431	37.18	2	Syndecan‐4 OS = Homo sapiens OX = 9606 GN = SDC4 PE = 1 SV = 2
PTPN23	Q9H3S7	28.84	2	Tyrosine‐protein phosphatase non‐receptor type 23 OS = Homo sapiens OX = 9606 GN = PTPN23 PE = 1 SV = 1
HEG1	Q9ULI3	26.59	2.04	Protein HEG homolog 1 OS = Homo sapiens OX = 9606 GN = HEG1 PE = 1 SV = 3
PACSIN3	Q9UKS6	44.39	2.09	Protein kinase C and casein kinase substrate in neurons protein 3 OS = Homo sapiens OX = 9606 GN = PACSIN3 PE = 1 SV = 2
GPRC5A	Q8NFJ5	37.27	2.14	Retinoic acid‐induced protein 3 OS = Homo sapiens OX = 9606 GN = GPRC5A PE = 1 SV = 2
ITCH	Q96J02	31.18	2.23	E3 ubiquitin‐protein ligase Itchy homolog OS = Homo sapiens OX = 9606 GN = ITCH PE = 1 SV = 2
NRP2	Q7LBX6	30.14	2.29	Neuropilin OS = Homo sapiens OX = 9606 GN = NRP2 PE = 1 SV = 1
PCDHGC3	Q9UN70	26.52	2.34	Protocadherin gamma‐C3 OS = Homo sapiens OX = 9606 GN = PCDHGC3 PE = 1 SV = 1
CD81	P60033	57.06	2.36	CD81 antigen OS = Homo sapiens OX = 9606 GN = CD81 PE = 1 SV = 1
PACSIN2	Q9UNF0	45.88	2.41	Protein kinase C and casein kinase substrate in neurons protein 2 OS = Homo sapiens OX = 9606 GN = PACSIN2 PE = 1 SV = 2
IGF2BP2	Q9Y6M1	36.41	2.43	Insulin‐like growth factor 2 mRNA‐binding protein 2 OS = Homo sapiens OX = 9606 GN = IGF2BP2 PE = 1 SV = 2
YTHDF1	Q9BYJ9	39.38	2.5	YTH domain‐containing family protein 1 OS = Homo sapiens OX = 9606 GN = YTHDF1 PE = 1 SV = 1
YTHDF3	Q7Z739	39.38	2.5	YTH domain‐containing family protein 3 OS = Homo sapiens OX = 9606 GN = YTHDF3 PE = 1 SV = 1
ATXN2L	H3BUF6	28.27	2.54	Ataxin‐2‐like protein OS = Homo sapiens OX = 9606 GN = ATXN2L PE = 1 SV = 1
CLTC	Q00610	65.9	2.59	Clathrin heavy chain 1 OS = Homo sapiens OX = 9606 GN = CLTC PE = 1 SV = 5
SF3B3	Q15393	31.74	2.61	Splicing factor 3B subunit 3 OS = Homo sapiens OX = 9606 GN = SF3B3 PE = 1 SV = 4
NOTCH2	Q04721	20.19	2.63	Neurogenic locus notch homolog protein 2 OS = Homo sapiens OX = 9606 GN = NOTCH2 PE = 1 SV = 3
LAYN	Q6UX15	25.62	2.71	Layilin OS = Homo sapiens OX = 9606 GN = LAYN PE = 2 SV = 1
PCBP1	Q15365	43.8	2.81	Poly(rC)‐binding protein 1 OS = Homo sapiens OX = 9606 GN = PCBP1 PE = 1 SV = 2
SNX33	Q8WV41	37.22	3.06	Sorting nexin‐33 OS = Homo sapiens OX = 9606 GN = SNX33 PE = 1 SV = 1
EDIL3	O43854	96.62	3.14	EGF‐like repeat and discoidin I‐like domain‐containing protein 3 OS = Homo sapiens OX = 9606 GN = EDIL3 PE = 1 SV = 1
CLTB	P09497	56.95	3.18	Clathrin light chain B OS = Homo sapiens OX = 9606 GN = CLTB PE = 1 SV = 1
CLTA	P09496	71.81	3.23	Clathrin light chain A OS = Homo sapiens OX = 9606 GN = CLTA PE = 1 SV = 1
PCBP2	Q15366	68.08	3.28	Poly(rC)‐binding protein 2 OS = Homo sapiens OX = 9606 GN = PCBP2 PE = 1 SV = 1
SH3GL1	Q99961	92.89	3.3	Endophilin‐A2 OS = Homo sapiens OX = 9606 GN = SH3GL1 PE = 1 SV = 1
SRP14	P37108	69	3.74	Signal recognition particle 14 kDa protein OS = Homo sapiens OX = 9606 GN = SRP14 PE = 1 SV = 2
PABPC1	P11940	107.16	3.86	Polyadenylate‐binding protein 1 OS = Homo sapiens OX = 9606 GN = PABPC1 PE = 1 SV = 2
GCN1	Q92616	26.34	4.21	eIF‐2‐alpha kinase activator GCN1 OS = Homo sapiens OX = 9606 GN = GCN1 PE = 1 SV = 6
CPSF6	Q16630	20.41	4.28	Cleavage and polyadenylation specificity factor subunit 6 OS = Homo sapiens OX = 9606 GN = CPSF6 PE = 1 SV = 2
SRP9	P49458	93.07	5.22	Signal recognition particle 9 kDa protein OS = Homo sapiens OX = 9606 GN = SRP9 PE = 1 SV = 2

Only those proteins quantitated with two unique peptides were considered. Spectrum filter and significance (20 and PEAKSQ method) was used. Data corresponding to CD63 (which is just below the 1.5 folds threshold) are also shown

To have further cues about the EVs in the secretome of CD9 KO cells, we performed MALDI‐MS lipidomic assays on membrane arrays of EV samples (Figure 4d). These analyses revealed a significant increase in different molecular forms of sphingomyelin (SM) in CD9 deleted EV compared to control samples, while some forms of phosphatidylcholine (PC) were significantly downregulated, reinforcing the notion of an overrepresentation of endosomal‐derived vesicles in CD9 KO cells secretome.

### 3.3 CD9 cytopermeable peptide reduces tumour cell growth in a xenograft model

**FIGURE 5:**
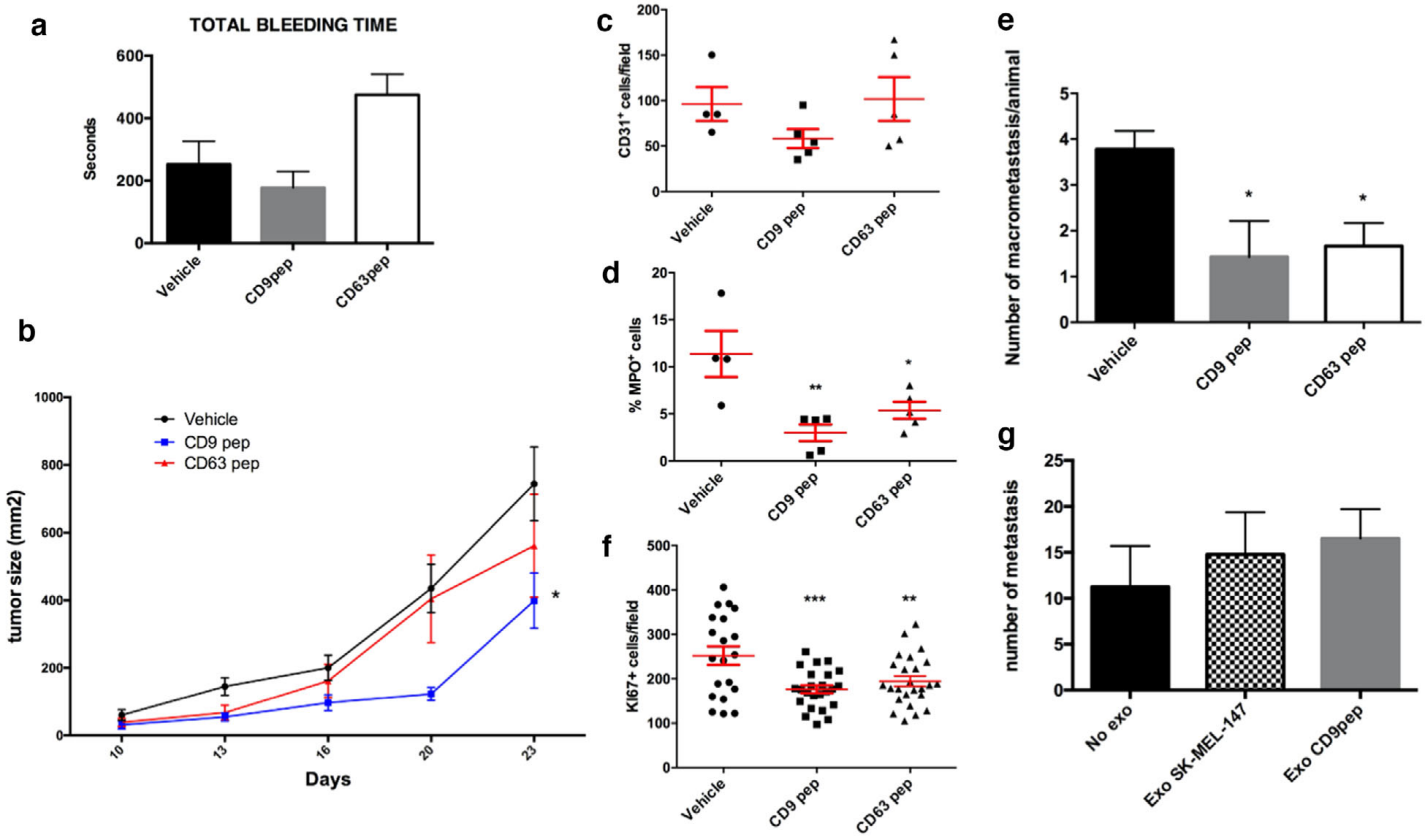
Analyses of the *in vivo* effects of tetraspanin cytopermeable peptides. a. Analysis of total tail bleeding time of mice treated with subcutaneous injections of vehicle, CD63 peptide or CD9 peptide n = 3. Data are represented as the mean ± SEM. b. Analysis of primary tumour size along 21 d subjected to serial intratumour injections of vehicle, CD63 peptide or CD9 peptide. Data correspond to two independent experiments each one performed with groups of n = 5 animals and are represented as the mean ± SEM. Data were statistically significant * *P* < 0.05 for CD9 peptide compared to vehicle in a two way ANOVA analysis with Dunnett's multiple comparisons post‐test. c. Analysis of tumour angiogenesis by immunohistochemical staining of CD31 endothelial marker. Data correspond to n = 5 represented as the mean ± SEM. d. Analysis of tumour infiltration by immunohistochemical staining of myeloperoxidase (MPO) to evaluate neutrophil infiltration. Data correspond to n = 5 represented as the mean ± SEM of the percentage of infiltration area * < 0.05 ** < 0.01 in a one‐way ANOVA analysis with Bonferroni's multiple comparisons post‐test. e. Analysis of the number of lung macrometastases 15d after resection of the primary tumour. Data correspond to two independent experiments each one performed with groups of n = 5 animals and are represented as the mean ± SEM. * < 0.05 in a one way ANOVA analysis with Dunnett's multiple comparisons post‐test. f. Analysis of the number of proliferating (Ki67^+^) cells. Five different tissue sections from each primary tumour of n = 5 animals are represented as the mean ± SEM. ** < 0.01 *** < 0.001 in a one way ANOVA analysis with Dunnett's multiple comparisons post‐test. g. Analysis of the number of lung macrometastases in animals pre‐treated with 7 doses of EVs from control cells or from cells treated with CD9 peptide before intravenous injection of melanoma cells. Data correspond to one experiment performed with groups of n = 4 animals and are represented as the mean ± SEM

Both CD9 and CD63 are important regulators of melanoma progression and metastasis (Fan et al., 2010; Kondoh et al., 1993; Si & Hersey, 1993), and EVs have been reported to play a fundamental role in tumour metastatic niche formation (Peinado et al., 2012). Changes observed in the cellular architecture and EV composition upon treatment with tetraspanin cytopermeable peptides could impose an effect in tumour progression and metastasis formation. However, the C‐terminal sequence is 100% conserved between human and mice, and both CD9 and CD63 are highly expressed in platelets, so we were concerned about major deleterious effects on coagulation upon peptide treatment *in vivo*. A safety pilot assessment was performed in a small number of animals that were subcutaneously injected 7 times (twice a week) with 30 μg of either CD9 or CD63 peptides or vehicle. No deleterious effect on the animals or macroscopic organ abnormalities were observed. Haemograms remained normal during the whole procedure with only some effects that did not reach statistical significance in the coagulation time, shorter in CD9 peptide‐treated animals and longer than control in the CD63 peptide‐treated group (Figure 5a). These mild effects on coagulation were not detected in those experiments in which peptides were administered intra‐tumour (not shown).

In a xenograft model, mice were subcutaneously injected with 2 × 10^5^ melanoma cells previously pre‐treated for 7d with cytopermeable peptides *ex vivo*, followed by intra‐tumour injections of the peptides twice a week until resection of the primary tumour. These analyses revealed a slight delay in primary tumour growth when treated with CD63 peptide and a significant decrease with CD9 peptide (Figure 5b), with no significant effects in tumour angiogenesis (Figure 5c) but a significant reduction in tumour infiltration, of both neutrophils (assessed by myeloperoxidase staining) and of total leukocytes (with CD45 staining) (Figure 5d and data not shown). All primary tumours were resected when reached a volume of 1300 mm^2^ and lung metastases were evaluated three weeks after. No significant differences were observed in the total number of metastatic foci, but a clear reduction in their size, reflected in a significant reduction in lung macrometastases number with both tetraspanin‐targeted peptides (Figure 5e). The reduction in size in both primary tumours and metastases suggest an impairment of tumour cell growth, which was confirmed by Ki67 staining in histological sections from tumours treated with CD9 peptide and in a lesser extent with CD63 peptide (Figure 5f).

To directly evaluate the effect of EVs in metastatic niche formation, we pre‐treated mice with seven injections during 3 weeks of 10 μg of SK‐MEL‐147 EVs enriched by serial ultracentrifugation from conditioned media of untreated cells or cells treated with the CD9 peptide, before intravenous injection of untreated melanoma cells. As previously described, pretreatment of mice with melanoma EVs slightly facilitated metastasis formation (Peinado et al., 2012), but no differences were observed among animals treated with SK‐MEL‐147 EVs versus those pre‐treated with EVs from conditioned media of CD9 pep‐treated cells (Figure 5g).

### 3.4 CD9 peptide affects mitochondrial activity, which is compensated in CD9 CRISPR/Cas9 gene deleted cells by a reduction of mitophagy

**FIGURE 6:**
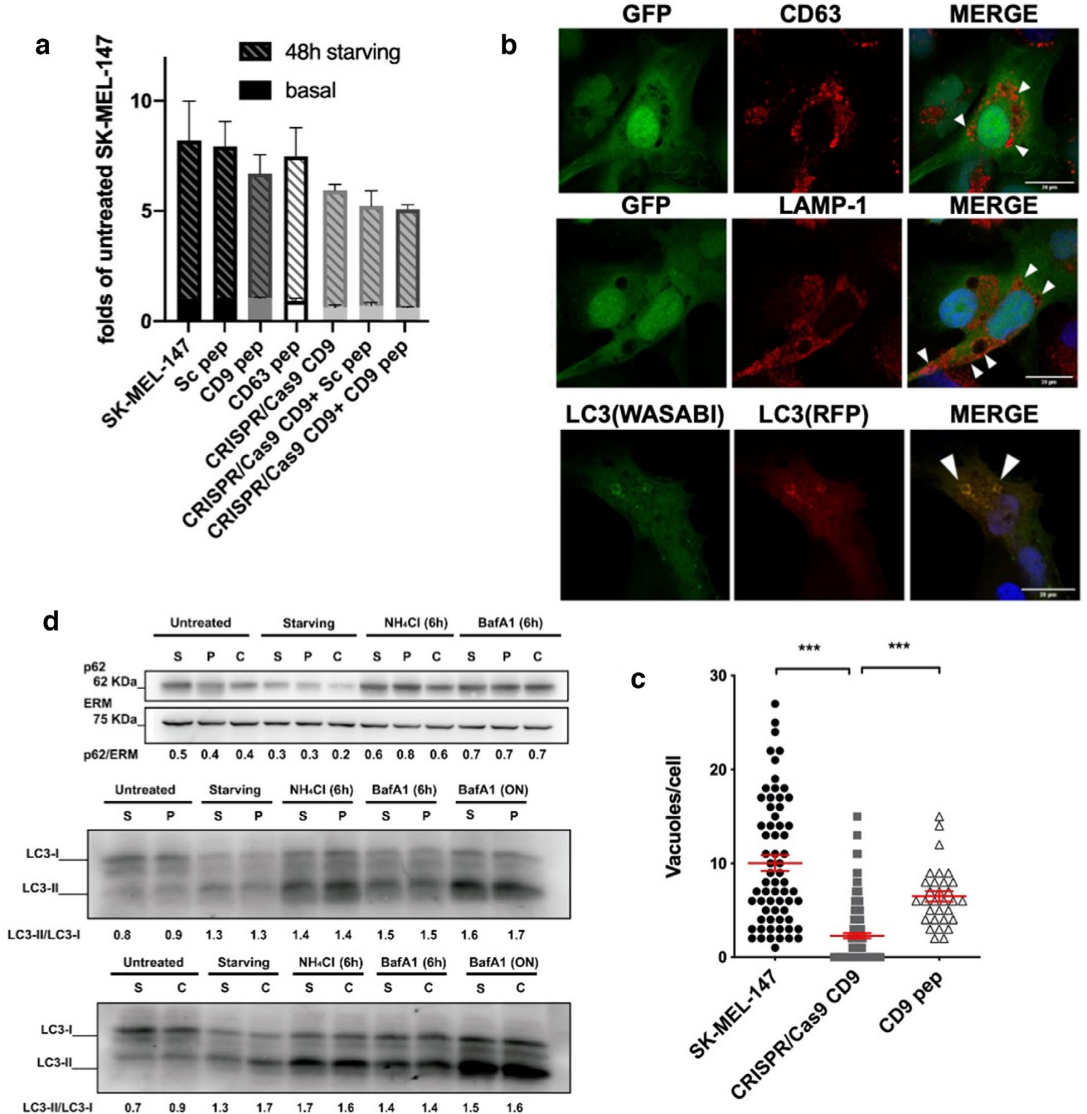
Effect of CD9 gene deletion or blockade on autophagy. a. Quantification of lysosomes by lysotracker staining by flow cytometry in normal culture conditions or after 48 h of serum starving. Data are represented as the mean ± SEM. b. To facilitate the observation and quantification of vacuoles, SK‐MEL‐147 cells were transfected with a plasmid encoding the GFP protein which is distributed homogeneously both in the cytoplasm and in the nucleus but did not gain access to large vacuoles in which no fluorescence was therefore detected and stained with antibodies specific for CD63 or LAMP‐1 and DAPI. Alternatively, cells were transfected with the LC3‐Wasabi/RFP construct. A maximal projection of a representative cell is shown. Arrowheads in the merge image point to large vacuoles. Bars = 20 μm c. Images of GFP‐transfected cells were acquired by confocal microscopy and the number of vacuoles per cell was quantified in a minimum of 30 cells/condition. Data are represented as the mean ± SEM. *** *P* < 0.001 in a one way ANOVA analysis with Dunnett's multiple comparisons post test. d. Total cell lysates of SK‐MEL‐147 cells untreated (S) or treated with CD9 peptide (P) or CRISPR/Cas9 CD9 gene‐deleted (C) were analysed by WB to assess p62 expression levels or LC3 lipidation. Cells were either left untreated or serum‐starved for 24h or pre‐treated for the indicated times with 50 mM NH_4_Cl or 100 nM bafilomycin A1. ERM was used as loading control. Numbers below the gels correspond to the densitometric analysis of the gels (p62/ERM or LC3II/LC3I)

The major effect observed upon treatment with CD9 peptide *in vivo* was a reduction in tumour cell proliferation. We could not detect alterations in cell cycle or apoptosis *in vitro* (not shown), so we decided to analyse endolysosomal acidification, autophagy flow and mitochondrial function. To assess endosomal acidification, we analysed lysotracker staining by flow cytometry. Although not very sensitive to quantitate the acidic compartment prevalence, some differences were observable in basal conditions, which mirrored those observed on electron microscopy micrographs (an increase in the mean fluorescence intensity in basal conditions of CD9 peptide‐ treated cells and a reduction in CRISPR/Cas9 CD9 KO cultures). When cells were subjected to starving conditions, a great increment in the fluorescence intensity of lysotracker staining was detected in all cultures, which was however, partially hampered in both CD9 peptide‐treated samples and CD9 KO cells (Figure 6a). We then analysed autophagy by LC3 staining. In these analyses we observed the presence of big vacuolar structures in these melanoma cells, which were greatly reduced in number in CD9 KO (Figure 6c) and, to a lower extent, upon CD9 peptide treatment (Figure 6c). These vacuoles were decorated by LC3 (Figure 6b) autophagosome marker but also stained with markers of late endosomes (HGS, CD63 and LAMP‐1) (Figure 6b and data not shown), and with tetraspanin CD9 in wild‐type cells (not shown). However, when we analysed autophagy flow by biochemical means, assessing both p62 expression and LC3 lipidation in normal and serum starving conditions, we could not detect differences between experimental groups, either with CD9 peptide treated cells or CD9 KO cells (Figure 6d). We included experimental conditions that inhibited lysosomal function with either NH_4_Cl or bafilomycin A1, which supported that lysosomal degradative activity was largely unaffected, since both LC3 and p62 accumulated upon lysosome degradation inhibition (Figure 6d).

**FIGURE 7:**
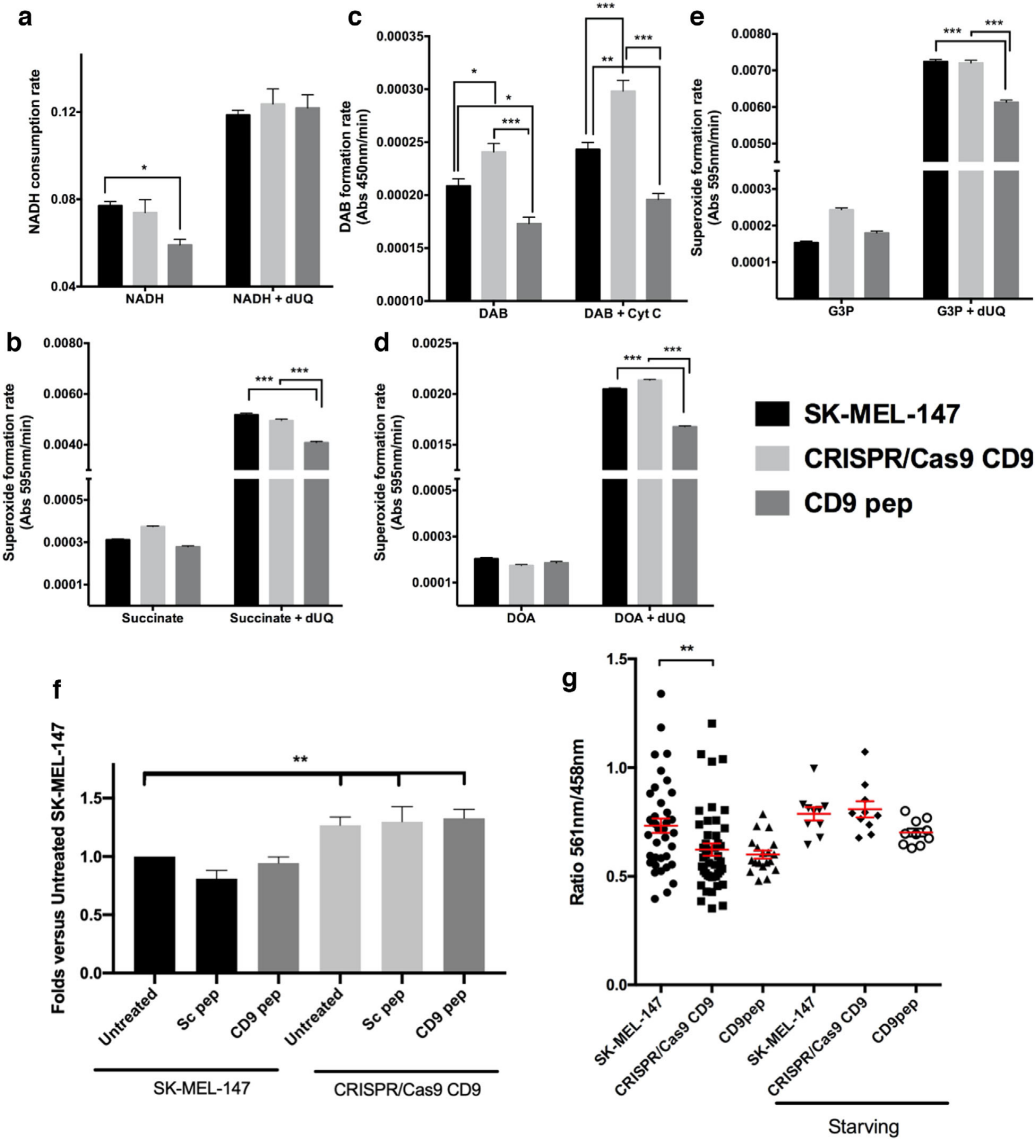
Effect of CD9 gene deletion or blockade on mitochondrial activity, number and mitophagy. a‐d. NADH dehydrogenase, Succinate dehydrogenase, Cytochrome c oxidase, Dihydroorotate dehydrogenase and Glycerol 3‐phosphate dehydrogenase activities were assessed on glass slide chips in which membrane homogenates from the different experimental conditions had been printed, in the absence and presence of 50 μM decylubiquinone (dUQ), when indicated; as described under methods. Analyses were performed in three experimental replicates by duplicated. Data are represented as the mean ± SEM. * *P* < 0.05, ** *P* < 0.01 and *** *P* < 0.001 in one way ANOVA analysis with Turkey's post‐test. f. Mean fluorescence intensity (MFI) after live‐cell staining with Mitotracker‐green probe was analysed by flow cytometry in SK‐MEL‐147 cells and CD9KO cells, untreated or treated with Sc or CD9 peptide. Data correspond to the mean ± SEM of normalized MFI (relative to untreated cells) of four independent experiments. ** *P* < 0.001 in one‐way ANOVA analysis. g. SK‐MEL‐147, treated or not with the CD9 peptide, and CD9 KO cells, were transfected with the mt‐mKeima construct and followed by live cell confocal microscopy. The relationship between the emission at 610 nm after excitation at either 561 nm or 458 nm is shown. Data are represented as the mean ± SEM. ** *P* < 0.01 in one‐way ANOVA analysis with Dunn´s post‐test

Since there is a functional connection of the endolysosomal apparatus and mitochondria (Audano et al., 2018), we decided to analyse mitochondrial activity on membrane homogenates from CD9pep‐treated and KO cells, printed onto glass slides using a non‐contact microarrayer. For Complex I activity, the rate of NADH consumption was determined and was shown to be significantly lower in cells treated with the CD9 cytopermeable peptide, and restored with dUQ, indicating a reduction in the Coenzyme Q content or in the affinity of the enzyme for this transporter (Figure 7a). Regarding Complex II, they presented a similar constitutive speed of succinate dehydrogenase activity (Figure 7b). However, in presence of dUQ, the rate of superoxide formation was significantly lower in the group treated with CD9 peptide. Similarly, cytochrome c oxidase activity was decreased in the group treated with the peptide, both in the absence and in the presence of exogenous cytochrome c (Figure 7c). We analysed superoxide formation with dihydroorotate and glycerol 3‐phosphate as substrates (Figure 7d and e), and were again significantly lower in the membranes of SK‐MEL cells treated with the peptide when the electron transporter is not a limiting factor. Surprisingly, in CD9 KO cells all these parameters of mitochondrial function were restored and we could only detect an increase in cytochrome c oxidase activity (Figure 7c).

To get further insights on the compensatory mechanisms behind the recovery of mitochondrial activity in CD9 KO cells, we quantitated total mitochondrial mass by flow cytometry analyses with Mitotracker Green probe (Figure 7f), which revealed a significant increment in CD9 KO cells. Malignant melanoma cells are greatly dependant on autophagy for maintaining their high proliferative rate (Checinska & Soengas, 2011). Autophagy is also related to mitochondria quality control. To specifically analyse the turnover of mitochondria by mitophagy, we transfected melanoma cells with a plasmid containing the sequence of the mitochondrial matrix protein COX VIII coupled to a mKeima fluorescent protein, that has a pH‐dependent fluorescent excitation (Sun et al., 2015). In these analyses, we observed a reduction of mitophagy in CD9 KO cells under normal culture conditions (Figure 7g). Upon starvation, mitophagy was increased in all cells so that in starving conditions there is no longer differences between wild‐type cells and CD9 KO cells. Changes induced by treatment with CD9 peptides showed the same tendency but did not reach significance.

Interestingly, these changes in mitophagy were reflected in the EV secretome of CD9 KO cells, in which the mitochondrial inner membrane serine hydroxymethyltransferase or the mitochondrial ATP synthase beta subunit were significantly reduced (Table 2 and Figure 4c). Other mitochondrial components were also underrepresented in these vesicles although changes did not reach the stringent threshold used for significance in the proteomic analyses (not shown). In addition, a reduction of components related to catabolic processes was also evident in the EV proteome (Supplementary Figure 6D, light blue).

## 4 DISCUSSION

Tetraspanins are a protein superfamily with 33 members in mammals, from which reliable antibodies have only been obtained for a few members. Because of their almost ubiquitous expression, CD63, CD9 and CD81 are the most commonly employed as EV markers, although other tetraspanins such as Tspan8 (Gesierich et al., 2006), CD37, CD82, CD53 (Escola et al., 1998) or CD151 (Yue et al., 2015) have also been reported on EVs from particular cell types. Tetraspanin abundancy is a common theme in extracellular vesicles, however, their functional role on EV biogenesis is still debated and there are conflicting results in the literature. For the most part, extracellular vesicle composition has been repeatedly shown to be altered upon tetraspanin gene deletion or silencing (Chairoungdua et al., 2010; Nazarenko et al., 2010; Perez‐Hernandez et al., 2013; Van Niel et al., 2011). More contradictory results have been described regarding EV number, with a reduction in total numbers (Chairoungdua et al., 2010; Hurwitz et al., 2016; Nazarenko et al., 2010; Perez‐Hernandez et al., 2013; Van Niel et al., 2011) or no effect (Brzozowski et al., 2018; Perez‐Gonzalez et al., 2012). In different tetraspanins, the C‐terminal sequence has been shown to be a crucial region for their interaction with several intracellular adaptor molecules and signalling cascades (Perez‐Hernandez et al., 2013). However, the flexibility at these cytosolic regions hampers their structural characterization by crystallographic approaches, so we decided to perform an ab initio modelling of the selected tetraspanins. Although the overlap of the full tetraspanin molecule from the ab initio model of CD81 with the crystalized structure was not complete, the overall structure was very similar, suggesting a good fitting outcome of this modelling approach. Some of the discrepancies observed between both structures may reside on the fact that the model derived from the crystal structure of CD81 is in the open conformation with a cholesterol molecule inserted in a pocket between the transmembrane regions. This conical open conformation may be in fact a requisite for tetraspanins incorporation and abundance on EV, which are highly curved membrane domains (Umeda et al., 2020). Our in silico approach allowed us to observe that the C‐terminal region of the different tetraspanins forms a bent structure in a loop facing the cytoplasm. When we then modelled the cell penetrating peptides used in our *in vitro* and *in vivo* studies, an interaction with the cytosolic surface of the membrane was observed to be dependent on the positively charged guanidinium groups in the Arginine stretch as previously described (Stanzl et al., 2013), which ensured a similar presentation of the sequence of the C‐terminal region of selected tetraspanins.

These peptides offer an experimental alternative approach to the study of tetraspanin function. Our previous studies in different cellular models showed a similar effect of the cytopermeable peptides to that observed upon gene silencing or deletion (Martínez Del Hoyo et al., 2015; Rocha‐Perugini et al., 2017; Tejera et al., 2013). Cytopermeable peptides are based on an arginine‐stretch motif that has been described that despite its polybasic nature, allows for a strong fork‐like interaction with the plasma membrane that leads to the adaptive translocation of the peptide across the membrane. This strategy is used by the Tat HIV protein and has been successfully adapted to several synthetic transporters (Stanzl et al., 2013). Once translocated, they will present the same structural conformation than the native molecule and are indeed incorporated into the endolysosomal pathway and secreted into EVs (not shown). Therefore, cytopermeable peptides are expected to interfere with tetraspanin intracellular interactome, which is quite complex (Perez‐Hernandez et al., 2013), so that the final functional outcome would be a sum of different effects, and determining which partner in the intracellular interactome of CD9 and CD63 C‐terminal sequence is involved in the phenotypes observed here will have to be further investigated in the future. Although complete functional overlap with gene deletion or silencing may not always occur. Tetraspanin gene deletion or silencing is in many cases compensated by an upregulation of the expression levels of other tetraspanin members. In fact, our data support, that even in early passages, CD9 was quickly compensated by CD63 expression upregulation. CD81 expression levels at the plasma membrane remained almost unchanged (not shown) although total cellular expression levels revealed by Western‐blot or flow cytometry on permeabilized samples were slightly reduced (Supplementary Figure 1C and D). Surprisingly, despite this fact, the incorporation of CD81 into EVs was moderately increased upon CD9 blockade or deletion, again supporting a functional compensatory role. In addition, these cytopermeable peptides would mostly affect those tetraspanin functions that rely on tetraspanin intracellular interactome, while molecular interactions via extracellular regions or lateral interactions with other transmembrane receptors may not be affected. Interestingly, expression of the targeted tetraspanin was required so no effect was observed for CD81 peptide on CD81‐null cells (Martínez Del Hoyo et al., 2015), nor in this report by treating CD9KO cells with CD9 peptide (Figures 6a, 7f, Supplementary Figure 5C), ruling out major off‐target effects of the peptides. All these results suggest that these cytopermeable peptides could offer an advantageous therapeutic tool to selectively block tetraspanin function, although, since they are dependent on the expression of the targeted tetraspanin, latest stages of melanoma, which usually downregulate both CD9 and CD63, may become refractory to treatment.

Both the treatment with the CD9 peptide and gene deletion impacted on the number of early endosomes. In contrast, different effects were observed in later maturation phases, being lysosomes increased by the CD9 peptide treatment while CD9 deletion increased MVB numbers. The latter effect could be explained by the compensatory upregulation of CD63 expression. This would suggest a specific role for each tetraspanin member in different phases of EV formation and release. Thus, CD9, which is expressed at high levels at the plasma membrane may relate to early endosome formation, CD63 would affect mostly at the MVB stage, while later steps in EV secretion may also be affected by other tetraspanins, such as recently demonstrated in Drosophila model, were tetraspanin Tsp96F overexpression reduces MVB targeting to the plasma membrane and thus EV release (González‐Méndez et al., 2020). Strikingly, despite the increased number of MVBs in CD9 KO cells, and their increased number of EVs secreted, those EVs seem to have a reduced protein cargo, since total protein measurements on samples obtained from the same number of cells revealed an increment in particle number but even a slight decrease in protein content. Different recent reports using mutants of CD63 endosomal localization motif (Fordjour et al., 2020; Mathieu et al., 2020) suggest that incorporation of tetraspanins into EVs is more effective when residing at the plasma membrane. Authors suggest that this effect can be explained by a more effective release by small microvesicles or ectosomes generation directly at the plasma membrane. Our data with CD9 KO cells, which have a reduced number of early endosomes and reduced protein content in the numerous EVs they release, suggest that an additional effect may reside in the trafficking from the plasma membrane through early endosomes, supporting that this may be a major route for EV protein cargo acquisition. However, small microvesicles may also be present in our EV samples, as reflected by the presence of histones, recently proposed to identify EVs derived from the plasma membrane or the amphisome route (Jeppesen et al., 2019). EV counting was performed directly on conditioned media by NTA and also confirmed with the Spectradyne system, based on resistive pulse sensing coupled to microfluidics. On this system 1.59 × 10^10^ particles/ml were quantified in the medium conditioned by SK‐MEL‐147 cells, 3.12 × 10^11^ particles/conditioned by ml in the medium of CRISPR/Cas9 CD9 cells and 2.2 × 10^10^ particles/ml in the cells treated with the CD9 peptide. Vesicles were also isolated by SEC and analysed by electron microscopy with similar results (Supplementary Figure 3). The increase in the relative content of sphingomyelin in EVs would also support an increment in endosomal derived vesicles secretion in CD9 KO cells. Similar increments in sphingomyelin were observed in vesicles recovered from conditioned media of CD9 peptide treated cells (not shown).

Changes in composition also suggest that tetraspanin peptides and CD9 gene deletion differentially affect EV subpopulations release. Both peptides impaired the secretion of different components that were reported to be present in a subpopulation of small EVs, namely the F5‐100K fraction (Kowal et al., 2016). However, in CD9 peptide treated or KO samples, we could detect an upregulation of CD81 and clathrin components. Tenascin, which has been recently described as a cargo of caveolin‐dependent EVs (Albacete‐Albacete et al., 2020) was upregulated by both peptides and CD9 gene deletion, while other extracellular matrix components have a differential behaviour in these samples.

Tetraspanins have been linked to tumour progression and metastasis by several mechanisms (Hemler, 2014; Ovalle et al., 2007; Schaper & Van Spriel, 2018), since they coordinate the function of adhesion receptors and enzymes at the plasma membrane forming tetraspanin‐enriched microdomains. CD9 has been reported to behave either as a tumour suppressor or promoter depending on the tumour studied (as most recent examples see (Li et al., 2020; Xing et al., 2020)). Both CD63 and CD9 play a role in melanoma progression and metastasis (Fan et al., 2010; Kondoh et al., 1993; Si & Hersey, 1993), although most classical reports were based on tetraspanin overexpression (Ikeyama et al., 1993). Our *in vivo* data demonstrated a decrease in primary tumour growth associated to a reduced proliferation that was more evident for CD9 peptide treatment, accompanied by a reduced tumour infiltration. However, no significant effect was observed on the number of metastases after intratumoral injection of our peptides, although their size was again smaller. In our experiment to directly evaluate metastatic niche formation based on pretreatment with EVs, we could not detect significant differences between animals pre‐treated with EVs derived from either CD9 peptide‐treated or untreated cells. Although we cannot completely rule out an effect on the competence of the metastatic niches formed by EVs secreted by cells treated with CD9 peptide, our data suggest that the major effect on tumour progression in this case could be attributed to an impairment in tumour cell proliferation.

Since we could not observe any effect on cell cycle or apoptosis, we decided to focus on those pathways that are linked to the endolysosomal compartment and that could be affecting the energetic metabolism, both autophagy and mitochondria. Autophagy is basally exacerbated in melanoma cells, being critical for maintaining their high proliferative rate (Checinska & Soengas, 2011). SK‐MEL‐147 cells showed prevalent vacuoles in their cytosol that were stained with both late endosomal markers and LC3, and that were reduced dramatically in CD9 KO cells and partially after treatment with CD9 peptide. To analyse the autophagy flow, we followed LC3 lipidation biochemically as well as LC3 incorporation into autophagosomes using a double‐tagged construct mTagRFP‐mWasabi‐LC3, based on the different stability of each of the fluorescent proteins at different pH so that in lysosomes only fluorescence from RFP is detected. We could observe an increment in LC3 dots that only emitted in the RFP channel upon fasting conditions in both CD9 KO cells and after CD9 peptide treatment (not shown). However, quantitation of this effect was hampered by the fact that LC3 is accumulated at different structures (big vacuoles and more discrete dots) in wt SK‐MEL‐147 cells compared to CD9 KO or CD9pep‐treated cells, where big vacuoles were decreased. Biochemical analyses revealed a functional autophagy and normal lysosomal function in CD9 KO cells. However, lysosomal increase during starvation seems to be compromised upon CD9 depletion or peptide treatment, despite the increment in basal levels of lysosomes observed in the case of CD9 peptide treatment. This may imply a delayed or worsen adaptability to metabolic changes in these cells. Overall, although starving‐induced autophagy seems largely unaffected in CD9 KO or CD9 peptide treated cells, less selective macroautophagy, may be affected upon CD9 gene deletion. All these data support a role for CD9 in lysosomal increment during starvation and in the formation of big LC3 positive degradative vacuoles in melanoma cells. Since LC3 translocation to endosomal structures has been recently proposed to be an ESCRT independent mechanism responsible for the sorting of some RNA‐binding proteins to the secretome (Leidal et al., 2020), it may be relevant to explore the connection of tetraspanin CD9 with this LC3‐depentent route for EV formation.

Changes in tumour cell proliferation may also be related to an effect on mitochondrial function induced by CD9 peptide treatment. Careful analyses of different enzymatic activities of mitochondrial complexes I (NADH dehydrogenase activity), II (Succinate dehydrogenase activity), and IV (Cytochrome c oxidase activity) (Dudkina et al., 2010) showed a reduced functionality in CD9 peptide‐treated samples. Because of the low speed of complex IV in SK‐MEL‐147 cells, complex III activity was not directly assessed by this method; although superoxide formation was also shown to be reduced upon CD9 peptide treatment. This impairment of mitochondrial complexes function upon CD9 peptide treatment may relate to a defect on mitochondrial turnover. SK‐MEL‐147 cells do not express parkin (Hu et al., 2016), so that mitophagy has to occur through an alternative route (Villa et al., 2018). To directly assess incorporation of mitochondria to acidic degradative compartments we used mt‐mKeima, again a pH sensitive tagged fluorescent molecule. In these assays, we could indeed detect a reduced mitophagy in CD9 peptide treated cells. In CD9 KO cells, the continuous reduction in mitophagy seems to be enough to compensate for the functional defects. An increment in mitochondrial mass was detected and overall enzymatic activities were restored, even presenting an increase in Cytochrome c oxidase activity. Although we could not detect significant changes in total cellular respiration by analysing oxygen consumption rate in a Seahorse equipment, CD9 KO cells had a reduced glycolytic activity as measured by lactate formation (not shown), suggesting that indeed mitochondrial activity may be enhanced in these cells.

One of the protein groups that are significantly altered in EVs from CD9 KO cells are mitochondrial components, further suggesting that EVs are part of the route for mitochondrial degradation at least in melanoma cells. The presence of mitochondrial components in EVs has been recently reported as a marker for melanoma and other tumours (Jang et al., 2019). In addition, routing mitochondrial components to EVs may serve as a danger signal for the induction of an immune response (Torralba et al., 2018). Thus, mitochondrial activity may be necessary to maintain EV secretion (Baixauli et al., 2015), but on the other hand, EV secretion may be crucial for mitochondrial homeostasis maintenance. The recovery of mitochondrial function may also allow the substantial increase in EV secretion observed in CD9 KO cells.

**FIGURE 8:**
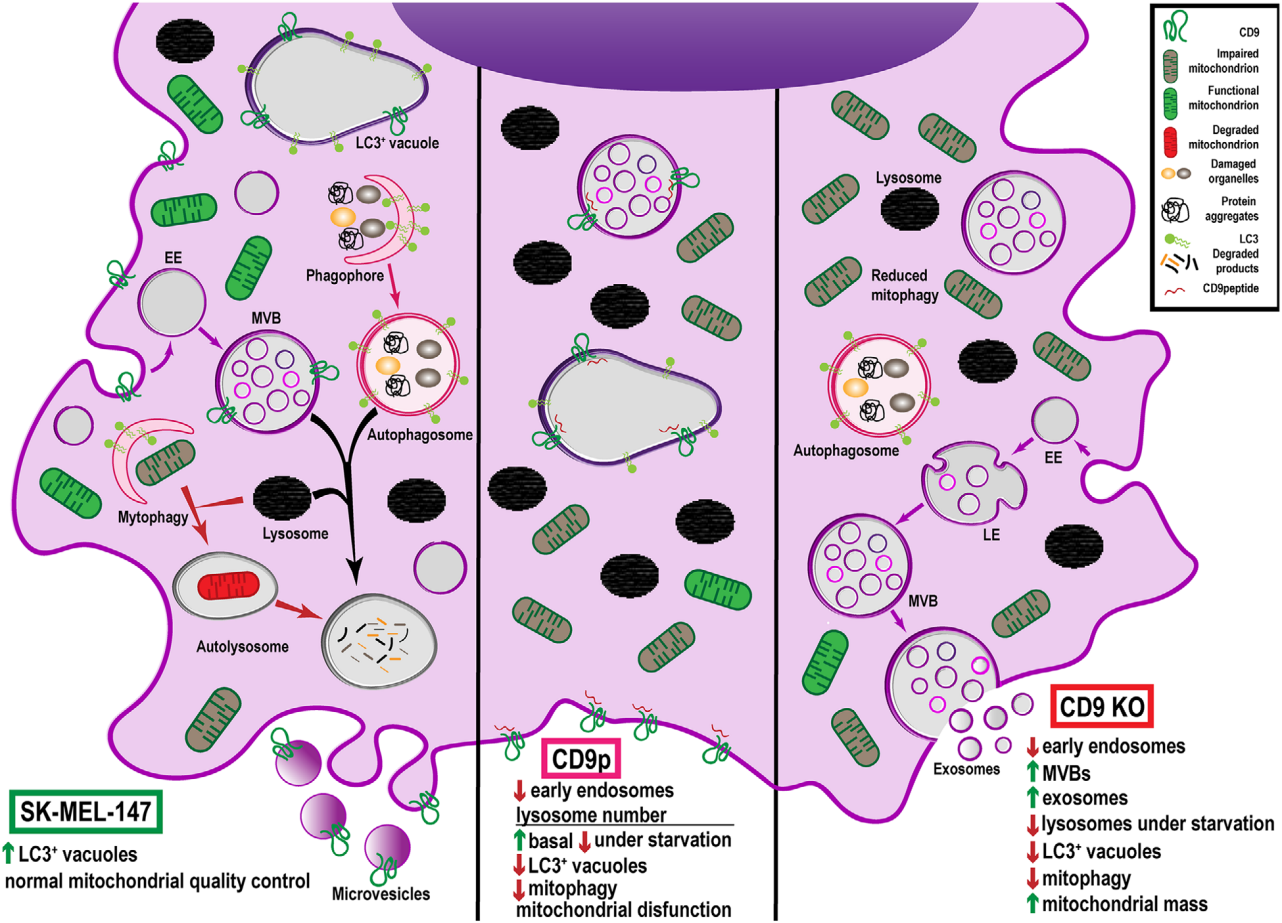
Model summarizing the effects observed in melanoma cells after treatment with CD9‐cytopermeable peptide or CD9 gene deletion

In summary, our data suggest that CD9 blockade by the cytopermeable peptide modifies the overall balance of the endolysosomal system (Figure 8). A reduction of early endosomes and vacuolar structures consistent with amphisomes would force an increase in lysosome formation to maintain proteolysis. However, this imbalance would impair mitochondrial turnover and quality control. When CD9 blockade is chronified by gene deletion, mitophagy reduction is able to increase overall mitochondrial mass and rescue functionality. Other compensatory mechanisms, such as the upregulation of different tetraspanin members, also take over, trying to ensure secretion of cytosolic components. Future studies should confirm whether these pathways are similarly regulated in other tumours and in non‐transformed cells.

## DECLARATION OF INTEREST STATEMENT

The authors report no conflict of interest.

## AUTHOR CONTRIBUTIONS

Henar Suárez, Zoraida Andreu, Carla Mazzeo and Víctor Toribio performed and analysed experimental data and figures; Aldo Emmanuel Pérez‐Rivera performed ab initio modelling, Soraya López‐Martín gave continuous technical support, Susana García‐Silva, Begoña Hurtado and Héctor Peinado provided support with mice experiments, Esperanza Morato, Laura Peláez and Ana Isabel Marina performed proteomic analyses, Egoitz Astigarraga Arribas, Tarson Tolentino‐Cortez and Gabriel Barreda‐Gómez performed mitochondrial and lipidomic analyses, María Yáñez‐Mó designed the experimental workflow and wrote the manuscript. All authors edited the manuscript.

## Supporting information

Supporting information.
