# Supplementary material for: CD9 inhibition reveals a functional connection of extracellular vesicle secretion with mitophagy in melanoma cells
Source: J Extracell Vesicles. 2021 May 12;10(7):e12082. doi: 10.1002/jev2.12082 (PMC8114031; doi:10.1002/jev2.12082)
Supplement: Supplementary file 1 — Supporting information. [file JEV2-10-e12082-s001.docx]

**
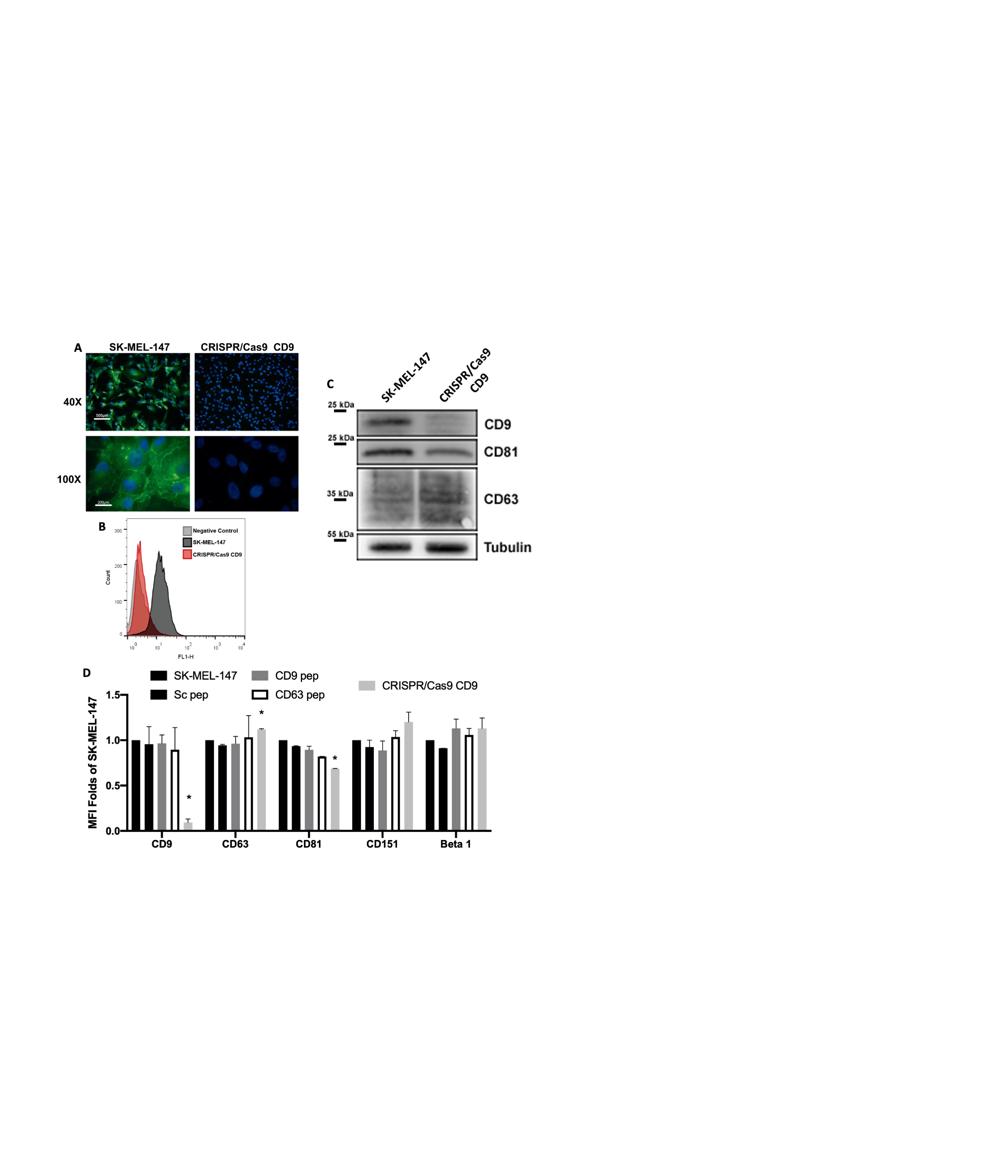
Supplementary Figure 1. A.** Analysis of CD9 expression in SK-MEL-147 cells after CD9 gene deletion by the CRISPR/Cas9 system by immunofluorescence staining with anti-CD9 VJ1/20 mAb, and confocal microscopy. Bars = 500 μm (40 X) and 200 μm (100X)**. B.** Analysis of CD9 expression in SK-MEL-147 cells after CD9 gene deletion by the CRISPR/Cas9 system by flow cytometry. **C.** Analysis of CD9 and tetraspanins CD81 and CD63 expression in SK-MEL-147 cells after CD9 gene deletion by the CRISPR/Cas9 system by western-blot in total cell lysates. Tubulin is shown as loading control. **D.** Analysis of CD9, tetraspanins CD63, CD81 and CD151 and integrin beta1 expression in CD9 KO SK-MEL-147 cells by flow cytometry. Data correspond to the mean ± SEM**. ***p< 0.05 in paired T-test respect to untreated SK-MEL-147.

**
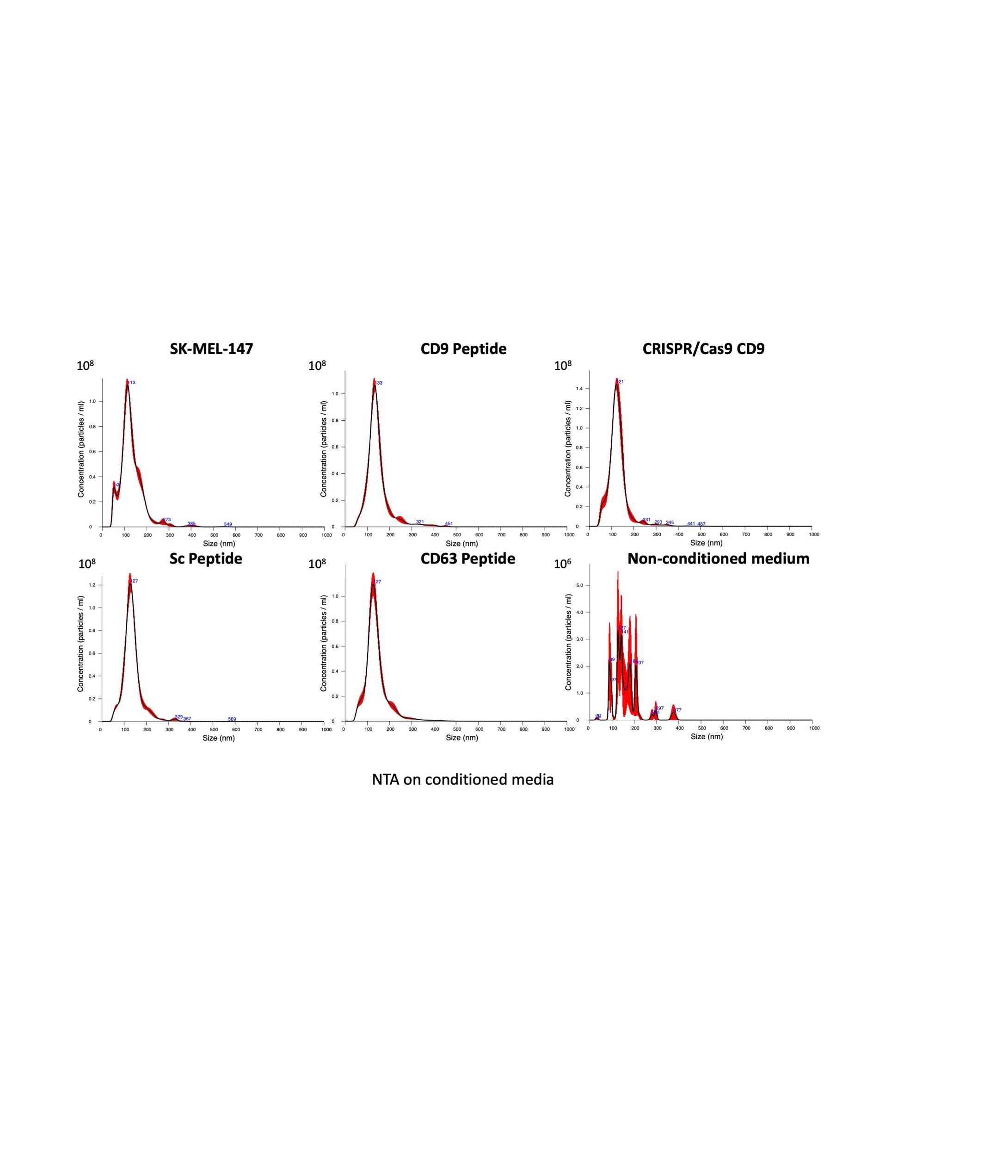
 Supplementary Figure 2.** Representative NTA profiles of 7d conditioned media in the indicated conditions. The logarithmic range is indicated on the axis. Unconditioned media was also analysed**.**


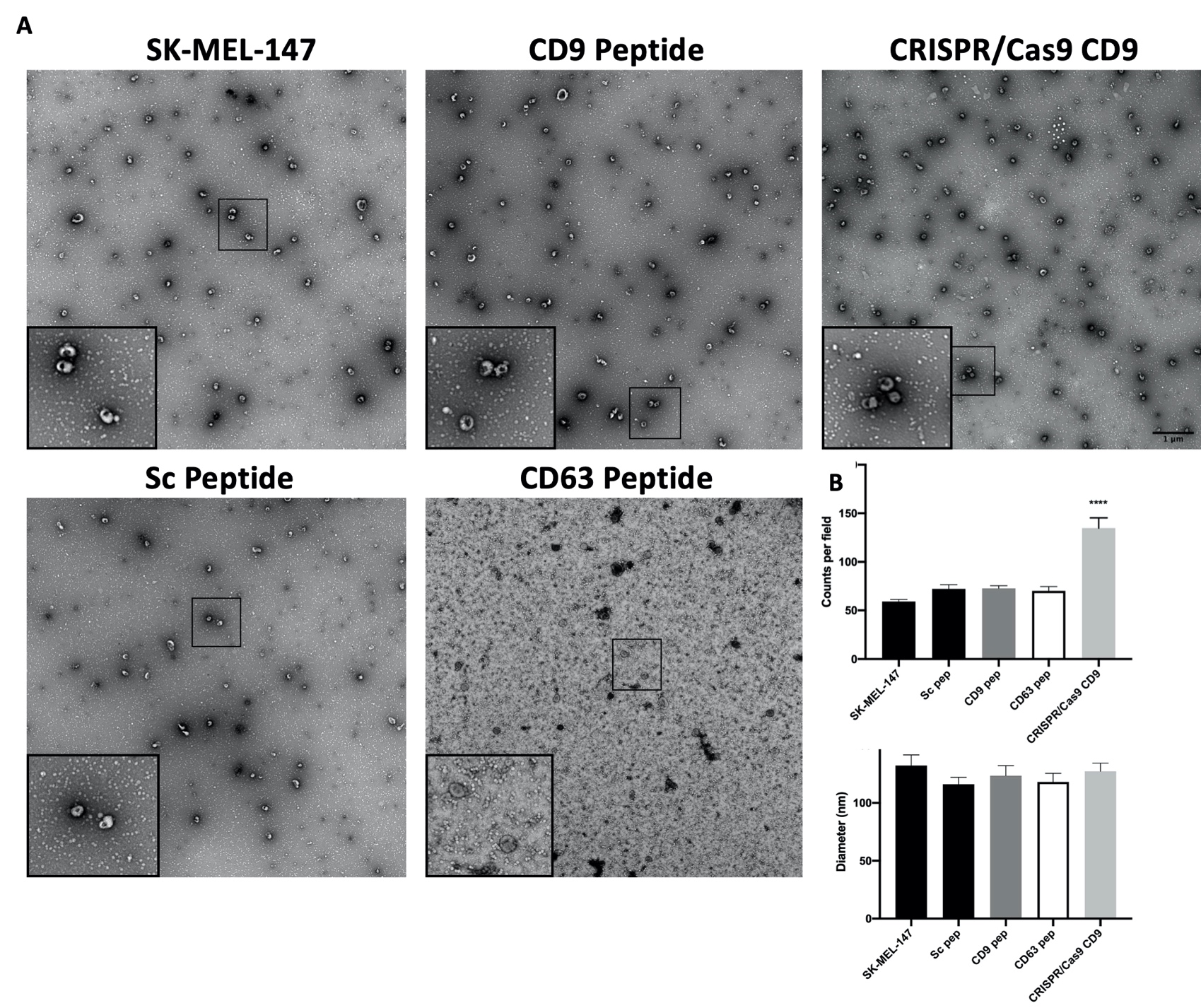


**Supplementary Figure 3. A.** Representative electron microscopy micrographs of negatively-stained SEC-isolated EV samples in the different culture conditions. Bar= 1 μm is shown in the CRISPR/Cas9 image. A close-up view of the marked area is shown in the insets. **B.** Images were quantitated using the TEM Exosome Analizer software ^47^. Data correspond to the mean ± SEM of a minimum of 20 fields from two independent experiments. ****p< 0.0001 in one-way ANOVA analysis respect to untreated SK-MEL-147.


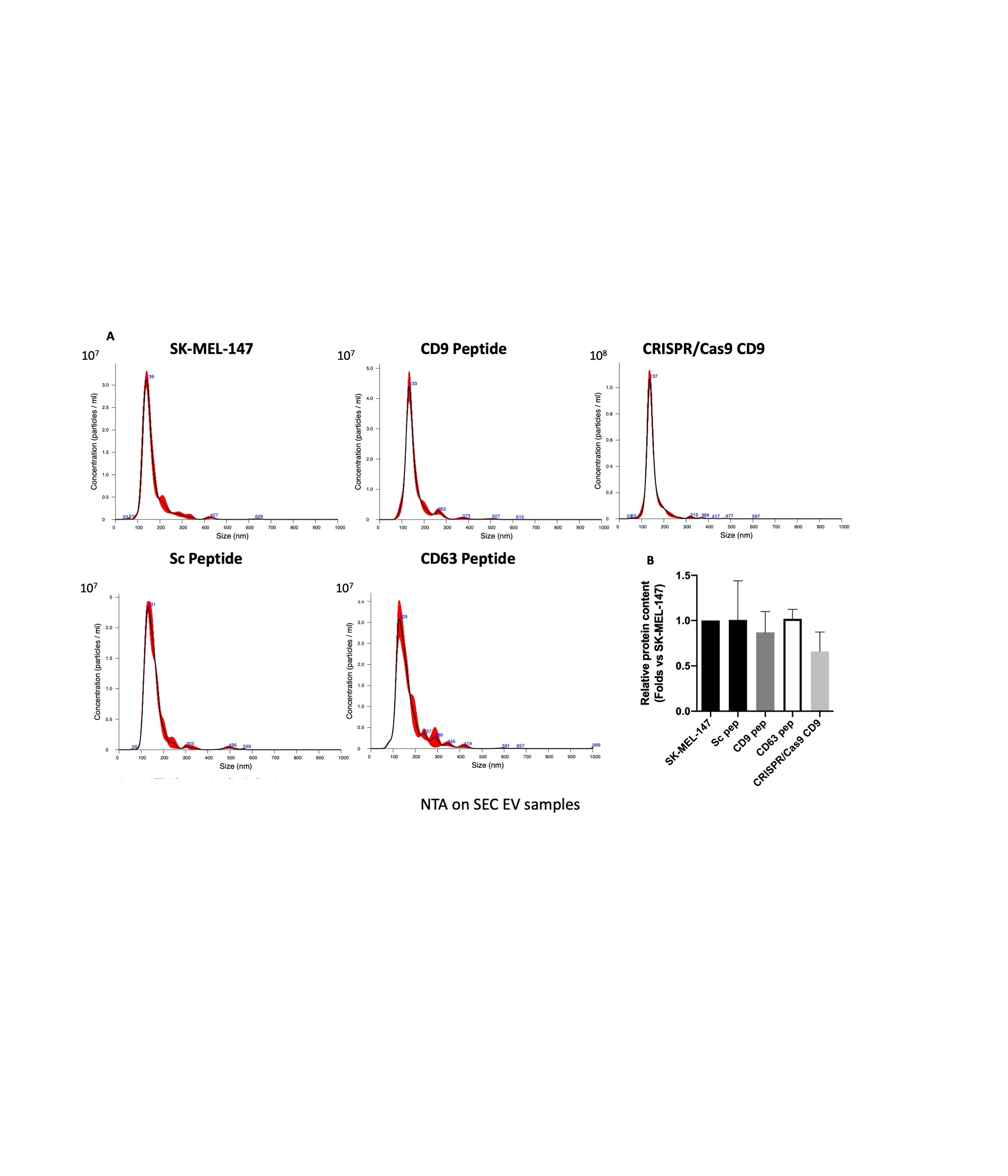


**Supplementary Figure 4. A.** Representative NTA profiles of SEC-isolated EV samples obtained from 7d conditioned media in the indicated conditions. The logarithmic range is indicated on the axis. **B.** BCA quantitation of the total protein content in SEC-isolated EV samples obtained from 7d conditioned media of the same number of cells in the indicated conditions. Data correspond to the mean ± SEM of three independent experiments.

**
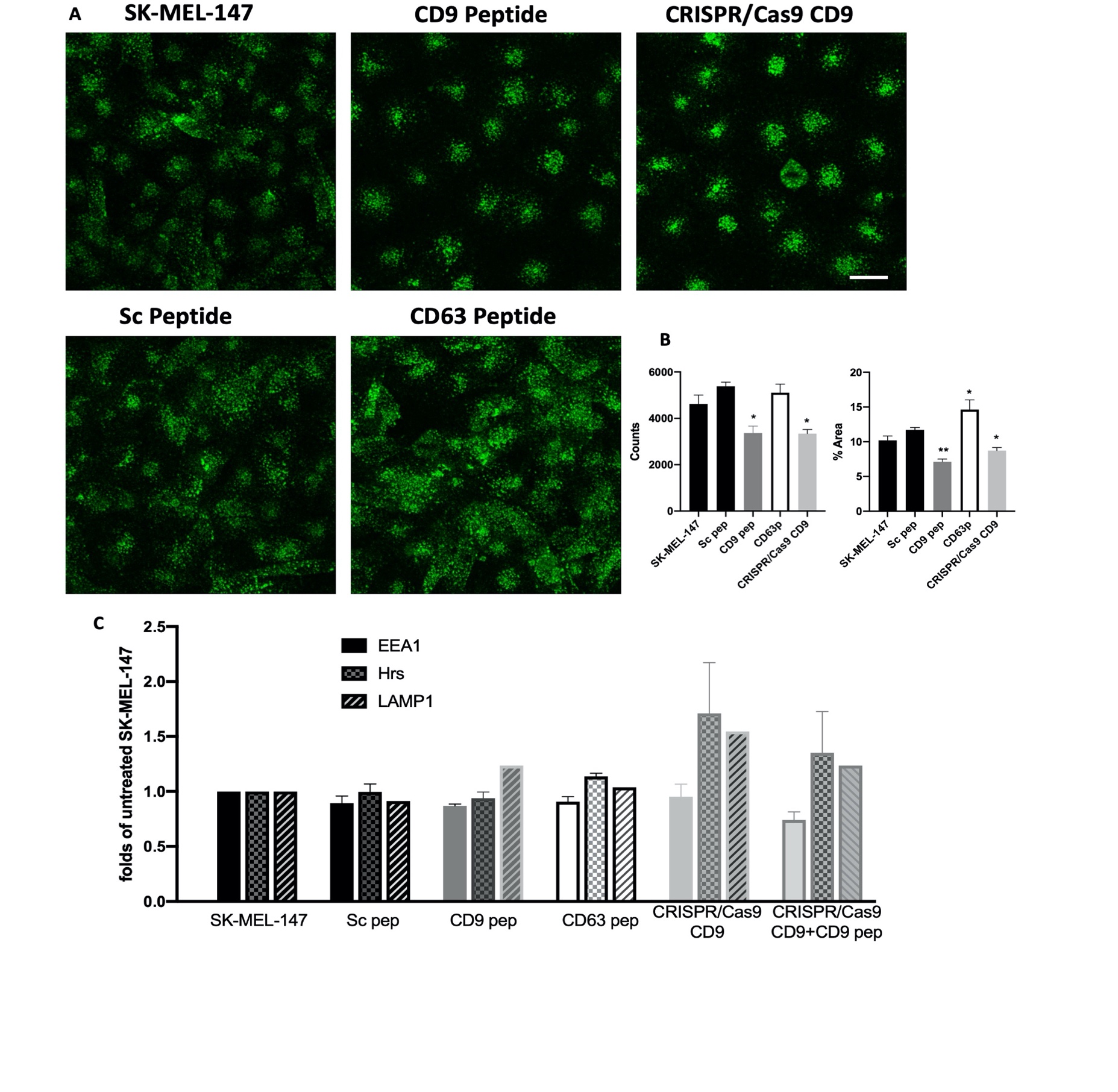
 Supplementary Figure 5. A.** SK-MEL-147 cells or CD9KO cells were cultured for 7d treated or not with the indicated peptides, fixed and stained with anti-EEA1 specific Ab. A maximal projection of a confocal stack is shown. Bar= 20 μm. **B.** Images were analysed with Image J by automatic thresholding. Plots depict the number of particles per field as well as the percentage of stained area. A reduction in the number of particles was significant in both CD9 peptide-treated cells and CD9 KO, as well as their perinuclear accumulation, so that the total stained area was also significantly reduced. Data correspond to the mean ± SEM. * p< 0.05, **p< 0.01 in Student T-test. **C.** Flow cytometry analyses of SK-MEL-147 cells or CD9KO cells, cultured for 7d treated or not with the indicated peptides. Samples were trypsinized, fixed and permeabilized before staining with specific Abs against EEA1, Hrs or LAMP1.

**
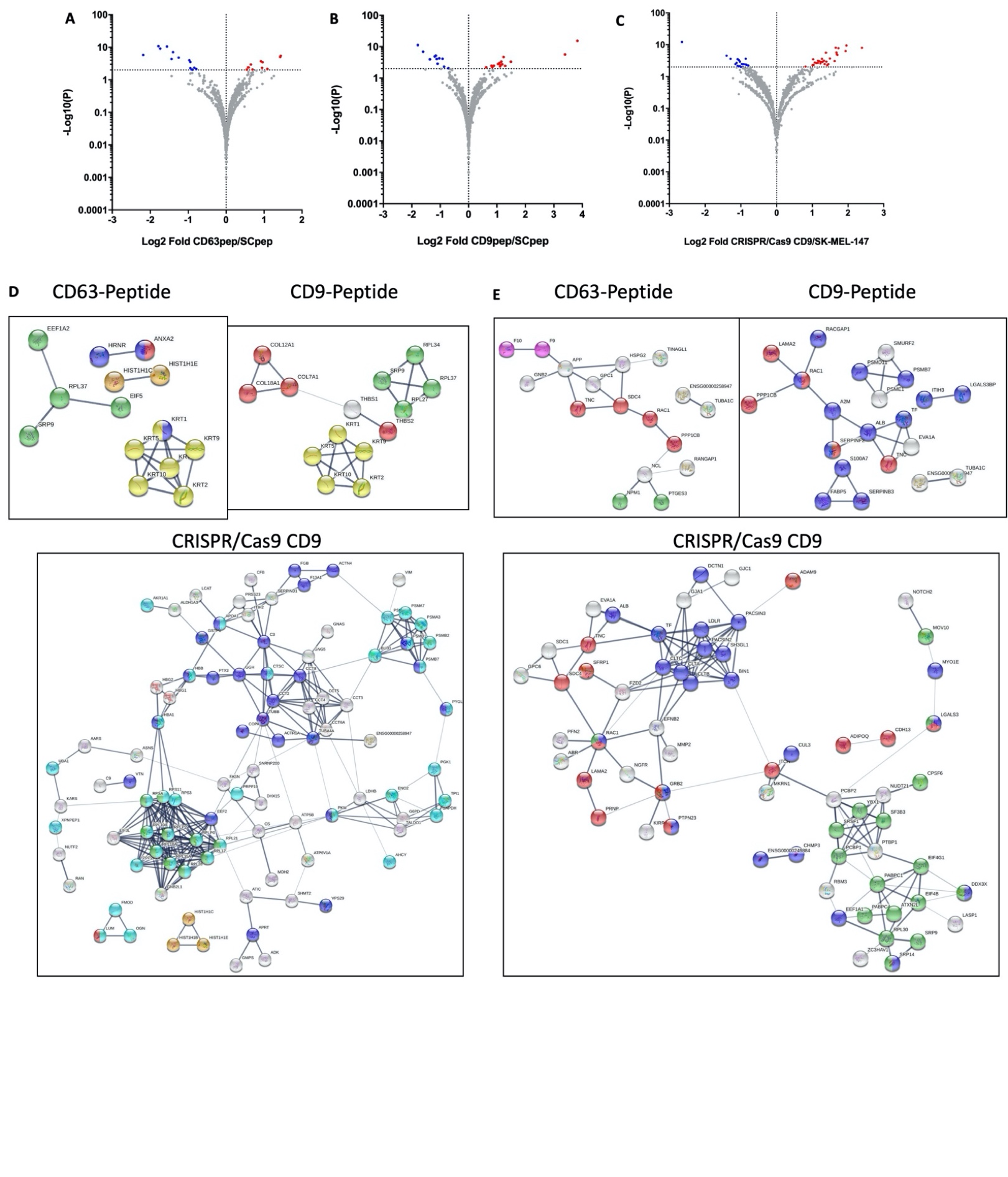
**

**Supplementary Figure 6.** Volcano plots of the distribution of the different proteins detected on SEC-isolated EVs from CD63 (**A**), CD9 (**B**) peptide-treated cells compared to control scramble peptide, or (**C**) from CD9 KO cells compared to wt SK-MEL-147. Horizontal line depicts the significance threshold used for Tables 1 and 2. STRING analyses of proteins downregulated (**D**) or upregulated (**E**) in SEC-isolated EV samples under the different conditions. Proteins related to vesicle trafficking are highlighted in blue, those related to cell adhesion in red. Keratins are depicted in yellow, histones in orange and ribosome or ribonucleoprotein components are depicted in green. Pink was used for coagulation-related proteins and light blue to those elements related to catabolic processes.
